# InDEx: Open Source iOS and Android Software for Self-Reporting and Monitoring of Alcohol Consumption

**DOI:** 10.5334/jors.207

**Published:** 2018-03-23

**Authors:** Daniel Leightley, Jo-Anne Puddephatt, Laura Goodwin, Roberto Rona, Nicola T. Fear

**Affiliations:** 1 King’s Centre for Military Health Research, Institute of Psychiatry, Psychology and Neuroscience, King’s College London, GB; 2 Department of Psychological Sciences, University of Liverpool, GB; 3 Academic Department of Military Mental Health, Institute of Psychiatry, Psychology and Neuroscience, King’s College London, GB

**Keywords:** alcohol, monitoring, smartphone, iOS, Android, IONIC

## Abstract

InDEx is a software package for reporting and monitoring alcohol consumption via a smartphone application. Consumption of alcohol is self-reported by the user, and the app provides a visual representation of drinking behaviour and offers feedback on consumption levels compared to the general population. InDEx is intended as an exemplar app, operating as a standalone smartphone application and is highly customisable for a variety of research domains. InDEx is written in JavaScript, using IONIC framework which is cross-platform and is available under the liberal GNU General Public License (v3). The software is available from GitHub (https://github.com/DrDanL/index-app-public).

## (1) Overview

### Introduction

Self-reported alcohol misuse is high amongst personnel of the United Kingdom (UK) Armed Forces, with the trend continuing after they leave service [1, 5]. More than 50% of those who have left military service meet the criteria for hazardous alcohol use, defined as scoring 8 or more on the Alcohol Use Disorders Identification Test [2, 3]. This prevalence rate is almost double that found in the general population [4]. Most people in the general population underestimate their drinking and do not perceive it as problematic, even when the level of consumption is potentially harmful to health [6]. This pattern is similar among Armed Forces personnel, with less than half of harmful drinkers recognising that they have an alcohol problem [7]. There is a culture of heavy alcohol use in the AF which may be encouraged or maintained by social determinants [8]; therefore, leaving service could provide an opportunity to initiate change in settings with less peer pressure to conform to social norms.

One medium in changing alcohol consumption is using a smartphone application (app). Existing alcohol apps emphasise longer-term health consequences which are seen as remote risks by young drinkers [9–11], however, a recent meta-analysis suggests that there may be greater benefits to focusing on shorter term consequences in order to encourage individuals to reduce their alcohol consumption [12, 13]. To the authors’ knowledge, we are unaware of any academic studies in this field which have released the source code under Open Source Initiative approved licenses to encourage reuse [14].

**Figure 1:**
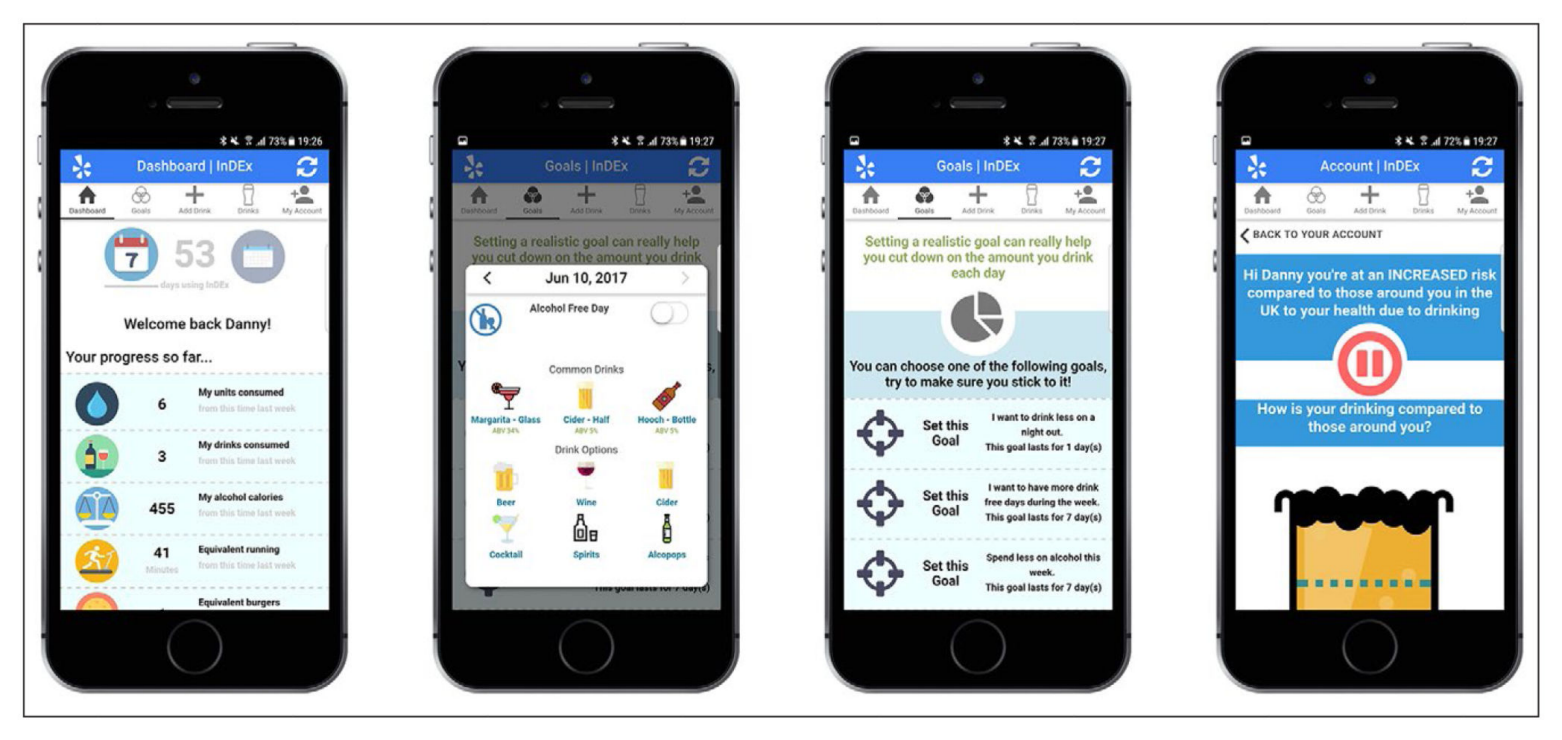
Example screenshots captured from the InDEx app.

**In**formation about **D**rinking for **Ex**-serving personnel (InDEx) was developed as a app to enable the self-reporting and monitoring of alcohol consumption in veterans who reside within the UK over a period of 28-days. See Figure 1 for screenshot examples of the InDEx app on a mobile device. InDEx was based on the following requirements:

- The app should be compatible across modern (released after 2012) Android (Google Inc, Mountain View, California, United States) and iOS (Apple Inc, Cupertino, California, United States) operating systems.
- The app should be capable of storing data locally and make it retrievable as required by the app.
- The app should allow users to self-report and record a range of alcoholic drinks (including quantity, who with and where) and offer the ability of recording a ‘Drink Free Day’.
- The app should be able to collect a range of self-reported measures obtained via in-app questionnaires.
- The system should provide a simple registration and signup process with minimal data collection.

The objective of this paper is to describe the development of the app. It will provide the research community with an exemplar app for use in other studies, and highlight the key stages to development.

### Implementation and architecture

InDEx was developed between October 2016 and March 2017 using JavaScript (ES6), HTML (5) and CSS (3).

#### Development life-cycle

**Figure 2:**
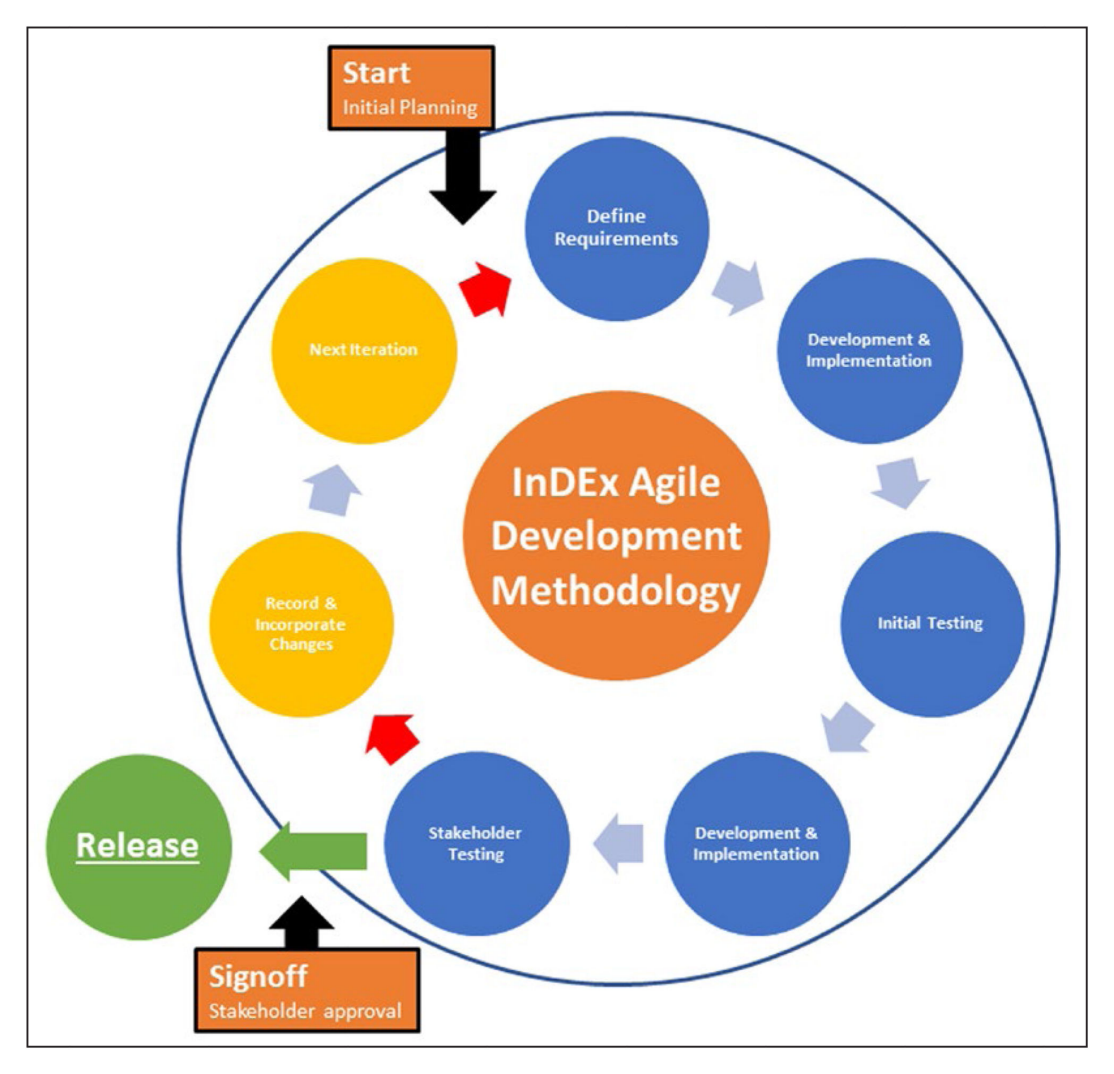
The Agile development methodology employed during development of the InDEx app.

InDEx used Agile development methodologies [15], with each cycle focusing firstly on the development and secondly on stakeholder/expert user testing (as illustrated in Figure 2). An incremental approach was employed, where each cycle built upon the functionality of the previous with new functionality introduced based on stakeholder/export user feedback. The cycle would not progress until stakeholder feedback on core features had been addressed.

#### Core Features

**Screening and Normative Feedback:** This module consists of two elements. First, at specific periods during the app life-cycle (*e.g.* day 0, 7, 14, 21 and 28) users are presented with a set of questionnaires (defined by the research team) and responses are logged (screening.js [state: screening]). Secondly, questionnaire responses are analysed to produce an informative visual feedback on alcohol consumption (normative.js [state: normative]).

**Alcohol Reporting and Monitoring:** This module consists of two elements. First, users are able to log alcohol consumption and ‘Drink Free Days’ (addDrink. js [state: adddrink]). Optional consent is available to obtain GPS location (geolocation.factory.js). Further, users can optionally record information on who they are with and where they are drinking. Second, a range of metrics are generated to provide an overview of current consumption (normative.controller.js [state: tabs/normative]).

**Goals:** This module enables users to set goal(s) based on implementation intentions [16]; a methodology that empowers the user to form self-regulation strategies in the form of an *if-then* plan. Users can select a goal (goals.js [state: tabs/goals]) and identify what is the biggest barrier to achieving that goal, Table 1 illustrates goal setting with *if-then*. Visual feedback is provided to users on progress towards achieving goal(s) and a status on those that have expired.

**Account Management:** This module (account.js [state: tabs/account]) enables users to modify and review personal information (*e.g.* first name, last name, mobile number), password and app parameters (*e.g.* automatic log out).

#### InDEx Implementation

The software is implemented using Drifty Co (Madison, Wisconsin, United States) IONIC Framework version 1, which is a cross-platform framework for web and mobile apps. The software has been implemented as a standalone application and does not require connection to a central service.

### Quality control

All functions have been individually tested for correctness to ensure their correct behaviour. Furthermore, all versions of the software underwent rigorous testing by stakeholder/expert users sourced from King’s Centre for Military Health Research and University of Liverpool (*n* = 17) to ensure software quality and usability.

## (2) Availability

### Operating system

Development: Windows 7 or above. Mac OS X El Capitan or above.

Production: Compatible on iOS and Android operating systems released after 2012.

### Programming language

InDEx is written in JavaScript (ES6), HTML5 and CSS3.

### Additional system requirements

None beyond requirements of the operating system and dependencies (listed hereafter).

### Dependencies

IONIC Framework version 1 (tested v3.0.0) [17]. All other dependencies are stated within the source code and accessed via a Content Delivery Network (CDN).

### List of contributors

This list of authors includes all main contributors. Daniel Leightley led the software development and is its current maintainer.

### Software location

#### Archive

***Name:*** Zenodo

***Persistent identifier:*** https://doi.org/10.5281/zendo.1068121

***Licence:*** GNU General Public License (v3)

***Publisher:*** Daniel Leightley

***Version published:*** 1.0.0

***Date published:*** 29/11/2017

#### *Code repository* GitHub

***Name:*** index-app-public

***Identifier:*** https://github.com/DrDanL/index-app-public

***Licence:*** GNU General Public License (v3)

***Date published:*** 29/11/2017

### Language

English (UK)

## (3) Reuse potential

InDEx (v1.0.0) enables research rapid access and reuse to an exemplar smartphone app for use in alcohol research, but provides the facility to modify the software for uses in other domains. The software has been made freely available to the community to further develop, extend and contribute to the app ecosystem. The GNU General Public License (v3) has been selected to ensure that developments are shared with the community, to the benefit of the community.

Support for modifying and using the software is available through GitHub issues page (https://github.com/DrDanL/index-app-public/issues) and GitHub wiki (https://github.com/DrDanL/index-app-public/wiki).

## Supplementary Material

Source Code

## Figures and Tables

**Table 1:** Example implementation intension goals for InDEx app.

Goal	Example *if-then*

I want to drink less on a night out	If other people are encouraging me to have a drink, then I will change my usual drink for a smaller measure
I want to have more drink free days during the week	If my partner is drinking at home then I will suggest that we have a set number of days to have a drink this week
I want to drink less overall this week	If I’m spending time with friends who drink a lot then I will start with water or a soft drink.
