# Supplementary material for: InDEx: Open Source iOS and Android Software for Self-Reporting and Monitoring of Alcohol Consumption
Source: J Open Res Softw. Author manuscript; Available in PMC 2018 May 21. (PMC5961935; doi:10.5334/jors.207)
Supplement: Source Code [file NIHMS77548-supplement-Source_Code.zip › www/lib/angular-chart.js/examples/charts.html]

Charts


Toggle navigation

Angular Charts

- Angular Chart
- Directives 
  - {{chart}}
- Getting started
- Reactive
- Mixed type charts


- Getting started
- Directives

# Angular Chart

Reactive, responsive, beautiful charts for AngularJS based on Chart.js

Code on Github

 Download (1.1.1)

# Getting started

### Dependencies

This repository contains a set of **native AngularJS directives** for Chart.js. The **only required dependencies** are:

- AngularJS (requires at least 1.4.x)
- Chart.js (requires Chart.js 2.x).

### Files to download

The easiest is to download with **npm**:

```
npm install angular-chart.js --save
```

Alternatively files can be downloaded from Github or via PolarArea.
See readme for more information.

Whichever method you choose the good news is that the overall size is very small:
<5kb for all directives (~1kb with gzip compression!)

### Installation

You need to include the dependencies in your page:

```
<script src="node_modules/chart.js/Chart.min.js"></script>
<script src="node_modules/angular-chart.js/dist/angular-chart.min.js"></script>
```

As soon as you've got all the files downloaded and included in your page you just need to declare
a dependency on the `chart.js` module:

```
angular.module('myModule', ['chart.js']);
```

### CSS

### Colors

Series have beautiful pre-sets colors (to a maximum of 7 series, after that colors will be randomly generated).
They can be overwritten using `Chart.defaults.global.colors`.

1. Blue
2. Light grey
3. Red
4. Green
5. Yellow
6. Grey
7. Dark Grey

You can also use the provider : `ChartJsProvider` in a `.config()`

```
(function (ChartJsProvider) {
  ChartJsProvider.setOptions({ colors : [ '#803690', '#00ADF9', '#DCDCDC', '#46BFBD', '#FDB45C', '#949FB1', '#4D5360'] });
});
```

# Directives

Line Chart

`.chart-line`

- `chart-data`: series data
- `chart-labels`: x axis labels
- `chart-options` (default: `{}`): Chart.js options
- `chart-series` (default: `[]`): series labels
- `chart-click` (optional): onclick event handler
- `chart-hover` (optional): onmousemove event handler
- `chart-colors` (default to global colors): colors for the chart
- `chart-dataset-override` (optional): override datasets individually


```
<canvas id="line" class="chart chart-line" chart-data="data"
chart-labels="labels" chart-series="series" chart-options="options"
chart-dataset-override="datasetOverride" chart-click="onClick">
</canvas>
```


```
angular.module("app", ["chart.js"]).controller("LineCtrl", function ($scope) {

  $scope.labels = ["January", "February", "March", "April", "May", "June", "July"];
  $scope.series = ['Series A', 'Series B'];
  $scope.data = [
    [65, 59, 80, 81, 56, 55, 40],
    [28, 48, 40, 19, 86, 27, 90]
  ];
  $scope.onClick = function (points, evt) {
    console.log(points, evt);
  };
  $scope.datasetOverride = [{ yAxisID: 'y-axis-1' }, { yAxisID: 'y-axis-2' }];
  $scope.options = {
    scales: {
      yAxes: [
        {
          id: 'y-axis-1',
          type: 'linear',
          display: true,
          position: 'left'
        },
        {
          id: 'y-axis-2',
          type: 'linear',
          display: true,
          position: 'right'
        }
      ]
    }
  };
});
```

`.chart-bar`

- `chart-data`: series data
- `chart-labels`: x axis labels
- `chart-options` (default: `{}`): Chart.js options
- `chart-series` (default: `[]`): series labels
- `chart-click` (optional): onclick event handler
- `chart-hover` (optional): onmousemove event handler
- `chart-colors` (default to global colors): colors for the chart
- `chart-dataset-override` (optional): override datasets individually


```
<canvas id="bar" class="chart chart-bar"
  chart-data="data" chart-labels="labels"> chart-series="series"
</canvas>
```


```
angular.module("app", ["chart.js"]).controller("BarCtrl", function ($scope) {
  $scope.labels = ['2006', '2007', '2008', '2009', '2010', '2011', '2012'];
  $scope.series = ['Series A', 'Series B'];

  $scope.data = [
    [65, 59, 80, 81, 56, 55, 40],
    [28, 48, 40, 19, 86, 27, 90]
  ];
});
```

Bar Chart

Doughnut Chart

`.chart-doughnut`

- `chart-data`: series data
- `chart-labels`: series labels
- `chart-options` (default: `{}`): Chart.js options
- `chart-click` (optional): onclick event handler
- `chart-hover` (optional): onmousemove event handler
- `chart-colors` (default to global colors): colors for the chart
- `chart-dataset-override` (optional): override datasets individually


```
<canvas id="doughnut" class="chart chart-doughnut"
  chart-data="data" chart-labels="labels">
</canvas>
```


```
angular.module("app", ["chart.js"]).controller("DoughnutCtrl", function ($scope) {
  $scope.labels = ["Download Sales", "In-Store Sales", "Mail-Order Sales"];
  $scope.data = [300, 500, 100];
});
```

`.chart-radar`

- `chart-data`: series data
- `chart-labels`: series labels
- `chart-options` (default: `{}`): Chart.js options
- `chart-series` (default: `[]`): series labels
- `chart-click` (optional): onclick event handler
- `chart-hover` (optional): onmousemove event handler
- `chart-colors` (default to global colors): colors for the chart
- `chart-dataset-override` (optional): override datasets individually


```
<canvas id="radar" class="chart chart-radar"
  chart-data="data" chart-options="options" chart-labels="labels">
</canvas>
```


```
angular.module("app", ["chart.js"]).controller("RadarCtrl", function ($scope) {
  $scope.labels =["Eating", "Drinking", "Sleeping", "Designing", "Coding", "Cycling", "Running"];

  $scope.data = [
    [65, 59, 90, 81, 56, 55, 40],
    [28, 48, 40, 19, 96, 27, 100]
  ];
});
```

Radar Chart

Pie Chart

`.chart-pie`

- `chart-data`: series data
- `chart-labels`: series labels
- `chart-options` (default: `{}`): Chart.js options
- `chart-click` (optional): onclick event handler
- `chart-hover` (optional): onmousemove event handler
- `chart-colors` (default to global colors): colors for the chart
- `chart-dataset-override` (optional): override datasets individually


```
<canvas id="pie" class="chart chart-pie"
  chart-data="data" chart-labels="labels" chart-options="options">
</canvas>
```


```
angular.module("app", ["chart.js"]).controller("PieCtrl", function ($scope) {
  $scope.labels = ["Download Sales", "In-Store Sales", "Mail-Order Sales"];
  $scope.data = [300, 500, 100];
});
```

`.chart-polar-area`

- `chart-data`: series data
- `chart-labels`: series labels
- `chart-options` (default: `{}`): Chart.js options
- `chart-click` (optional): onclick event handler
- `chart-hover` (optional): onmousemove event handler
- `chart-colors` (default to global colors): colors for the chart
- `chart-dataset-override` (optional): override datasets individually


```
<canvas id="polar-area" class="chart chart-polar-area"
  chart-data="data" chart-labels="labels" chart-options="options">
</canvas>
```


```
angular.module("app", ["chart.js"]).controller("PolarAreaCtrl", function ($scope) {
  $scope.labels = ["Download Sales", "In-Store Sales", "Mail-Order Sales", "Tele Sales", "Corporate Sales"];
  $scope.data = [300, 500, 100, 40, 120];
});
```

Polar Area Chart

Horizontal Bar Chart

`.chart-horizontal-bar`

- `chart-data`: series data
- `chart-labels`: x axis labels
- `chart-options` (default: `{}`): Chart.js options
- `chart-series` (default: `[]`): series labels
- `chart-click` (optional): onclick event handler
- `chart-hover` (optional): onmousemove event handler
- `chart-colors` (default to global colors): colors for the chart
- `chart-dataset-override` (optional): override datasets individually


```
<canvas id="base" class="chart-horizontal-bar"
  chart-data="data" chart-labels="labels" >
</canvas>
```


```
angular.module("app", ["chart.js"]).controller("BarCtrl",
  function ($scope) {
    $scope.labels = ['2006', '2007', '2008', '2009', '2010', '2011', '2012'];
    $scope.series = ['Series A', 'Series B'];

    $scope.data = [
      [65, 59, 80, 81, 56, 55, 40],
      [28, 48, 40, 19, 86, 27, 90]
    ];
});
```

`.chart-bubble`

- `chart-data`: series data
- `chart-labels`: x axis labels
- `chart-options` (default: `{}`): Chart.js options
- `chart-series` (default: `[]`): series labels
- `chart-click` (optional): onclick event handler
- `chart-hover` (optional): onmousemove event handler
- `chart-colors` (default to global colors): colors for the chart
- `chart-dataset-override` (optional): override datasets individually


```
<canvas id="base" class="chart-bubble" chart-data="data"
  chart-series="series" >
</canvas>
```


```
angular.module("app", ["chart.js"]).controller("BubbleCtrl",
  function ($scope) {
    // see examples/bubble.js for random bubbles source code
    $scope.series = ['Series A', 'Series B'];

    $scope.data = [
      [{
        x: 40,
        y: 10,
        r: 20
      }],
      [{
        x: 10,
        y: 40,
        r: 50
      }]
    ];
});
```

Bubble Chart

Dynamic Chart

Toggle

`.chart-base`

- `chart-type`: chart type e.g. bar, polarArea, etc. or other plugins
- other options according to chart type


```
<canvas id="base" class="chart-base" chart-type="type"
  chart-data="data" chart-labels="labels" >
</canvas>
```


```
angular.module("app", ["chart.js"]).controller("BaseCtrl",
  function ($scope) {
    $scope.labels = ["Download Sales", "In-Store Sales", "Mail-Order Sales", "Tele Sales", "Corporate Sales"];
    $scope.data = [300, 500, 100, 40, 120];
    $scope.type = 'polarArea';

    $scope.toggle = function () {
      $scope.type = $scope.type === 'polarArea' ?
        'pie' : 'polarArea';
    };
});
```

# Reactive

All charts are reactive and will update automatically when data changes.

Reactive Chart

Chart Data

| {{label}} |
| --- |
| {{data[$parent.$index][$index]}} |

Randomize

# Mixed type charts

It is possible to combine multiple types of charts on the same canvas e.g. bar and line.

```
<canvas id="base" class="chart-bar"
  chart-data="data" chart-labels="labels" chart-colors="colors"
  chart-dataset-override="datasetOverride" >
</canvas>
```


```
angular.module("app", ["chart.js"]).controller("MixedChartCtrl",
  function ($scope) {
    $scope.colors = ['#45b7cd', '#ff6384', '#ff8e72'];

    $scope.labels = ['Monday', 'Tuesday', 'Wednesday', 'Thursday', 'Friday', 'Saturday', 'Sunday'];
    $scope.data = [
      [65, -59, 80, 81, -56, 55, -40],
      [28, 48, -40, 19, 86, 27, 90]
    ];
    $scope.datasetOverride = [
      {
        label: "Bar chart",
        borderWidth: 1,
        type: 'bar'
      },
      {
        label: "Line chart",
        borderWidth: 3,
        hoverBackgroundColor: "rgba(255,99,132,0.4)",
        hoverBorderColor: "rgba(255,99,132,1)",
        type: 'line'
      }
    ];
});
```

Bar line Chart

Designed and built by Jerome Touffe-Blin

Code licensed under BSD License.

Issues

**Credits**: Chart.js and AngularJS
