# Supplementary material for: InDEx: Open Source iOS and Android Software for Self-Reporting and Monitoring of Alcohol Consumption
Source: J Open Res Softw. Author manuscript; Available in PMC 2018 May 21. (PMC5961935; doi:10.5334/jors.207)
Supplement: Source Code [file NIHMS77548-supplement-Source_Code.zip › www/lib/angular-chart.js/examples/tables.html]

Data tables


Chart Data

| {{label}} |
| --- |
| {{data[$parent.$index][$index]}} |

Reactive Chart
