# Supplementary figures and images for: InDEx: Open Source iOS and Android Software for Self-Reporting and Monitoring of Alcohol Consumption
Source: J Open Res Softw. Author manuscript; Available in PMC 2018 May 21. (PMC5961935; doi:10.5334/jors.207)

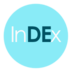

Supplement: Source Code [file NIHMS77548-supplement-Source_Code.zip › resources/android/icon/drawable-hdpi-icon.png]

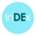

Supplement: Source Code [file NIHMS77548-supplement-Source_Code.zip › resources/android/icon/drawable-ldpi-icon.png]

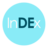

Supplement: Source Code [file NIHMS77548-supplement-Source_Code.zip › resources/android/icon/drawable-mdpi-icon.png]

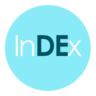

Supplement: Source Code [file NIHMS77548-supplement-Source_Code.zip › resources/android/icon/drawable-xhdpi-icon.png]

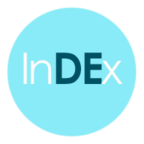

Supplement: Source Code [file NIHMS77548-supplement-Source_Code.zip › resources/android/icon/drawable-xxhdpi-icon.png]

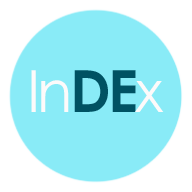

Supplement: Source Code [file NIHMS77548-supplement-Source_Code.zip › resources/android/icon/drawable-xxxhdpi-icon.png]

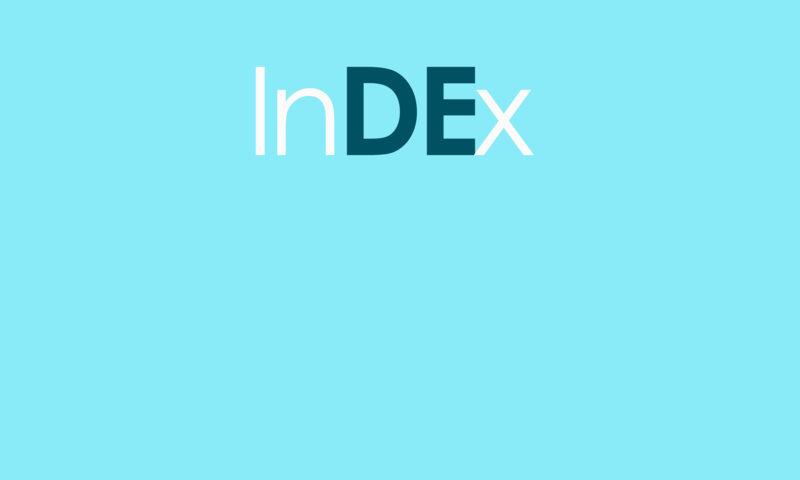

Supplement: Source Code [file NIHMS77548-supplement-Source_Code.zip › resources/android/splash/drawable-land-hdpi-screen.png]

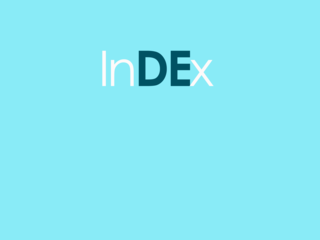

Supplement: Source Code [file NIHMS77548-supplement-Source_Code.zip › resources/android/splash/drawable-land-ldpi-screen.png]

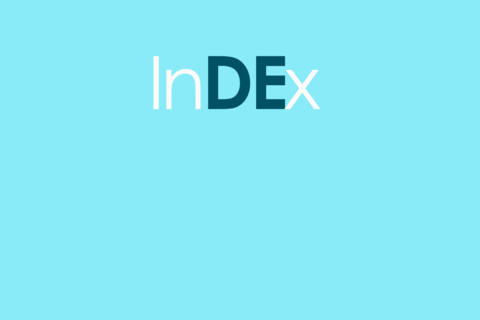

Supplement: Source Code [file NIHMS77548-supplement-Source_Code.zip › resources/android/splash/drawable-land-mdpi-screen.png]

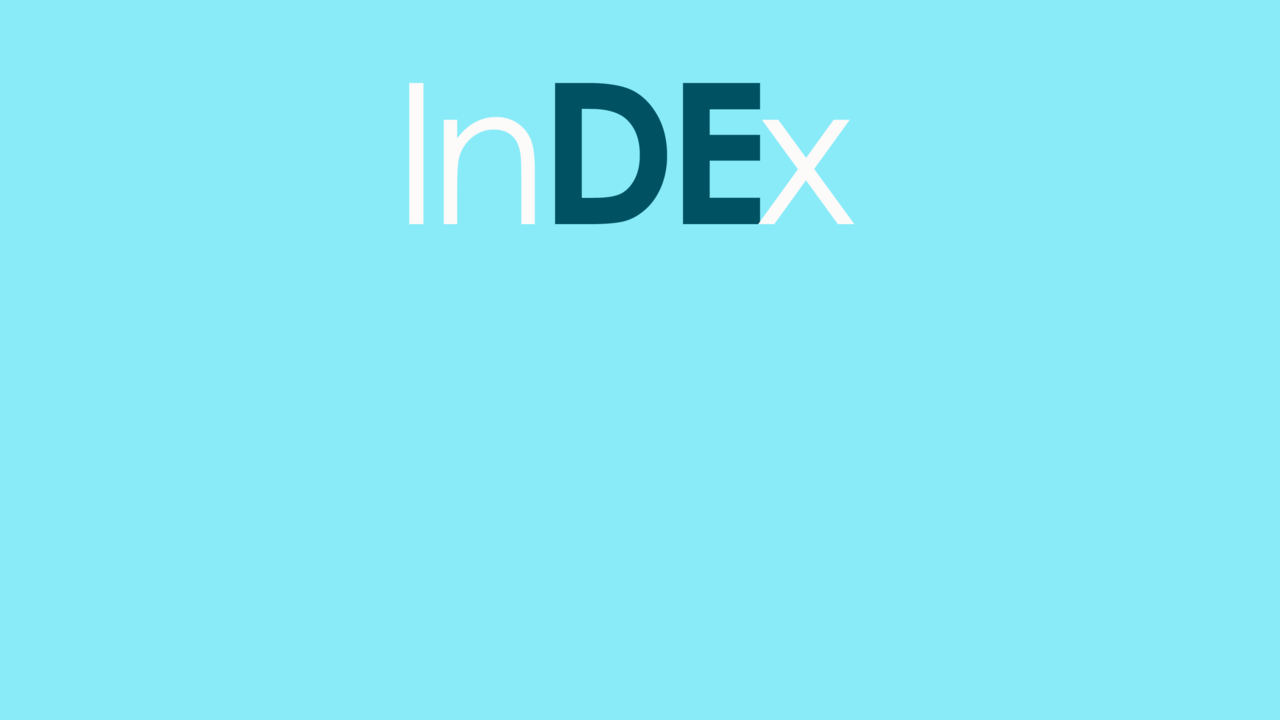

Supplement: Source Code [file NIHMS77548-supplement-Source_Code.zip › resources/android/splash/drawable-land-xhdpi-screen.png]

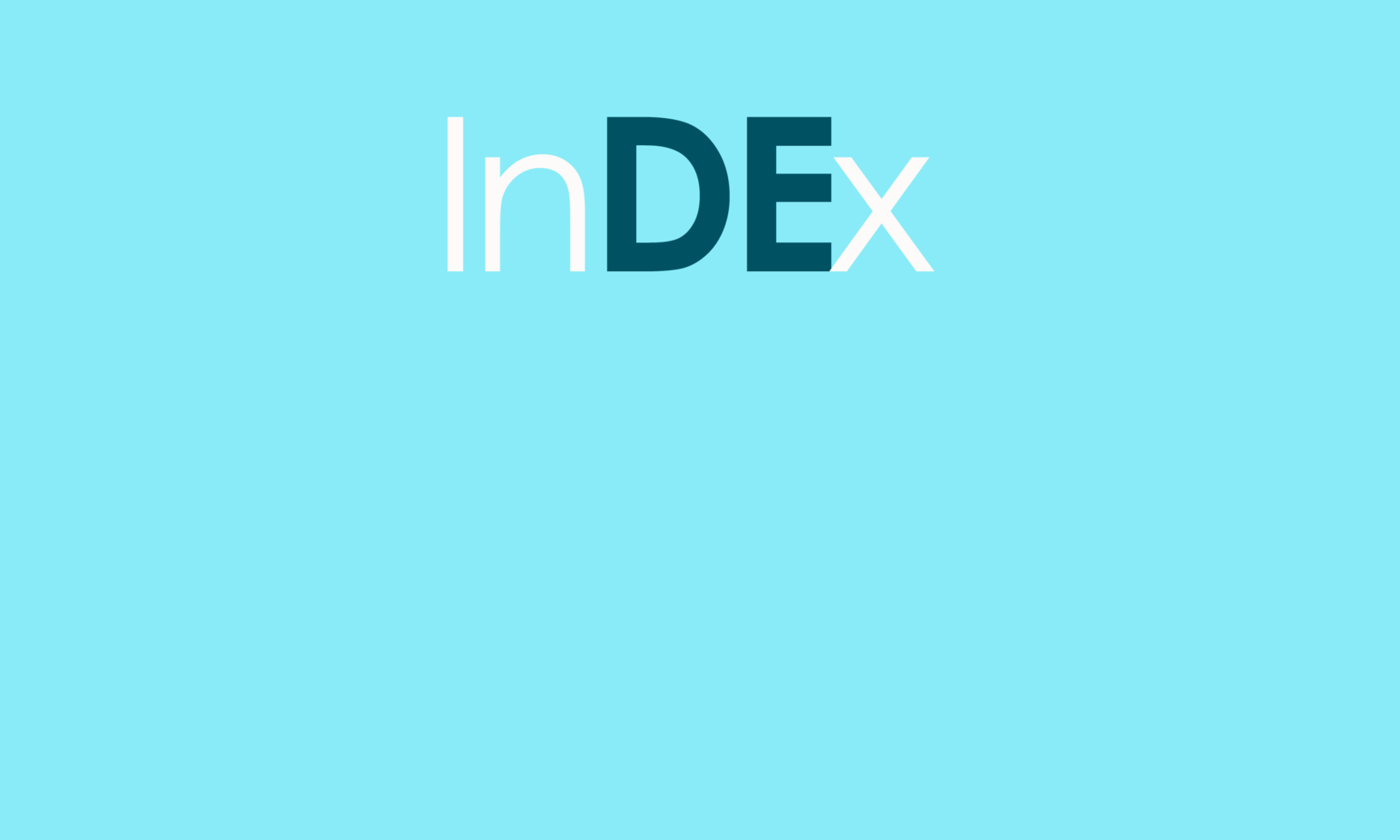

Supplement: Source Code [file NIHMS77548-supplement-Source_Code.zip › resources/android/splash/drawable-land-xxhdpi-screen.png]

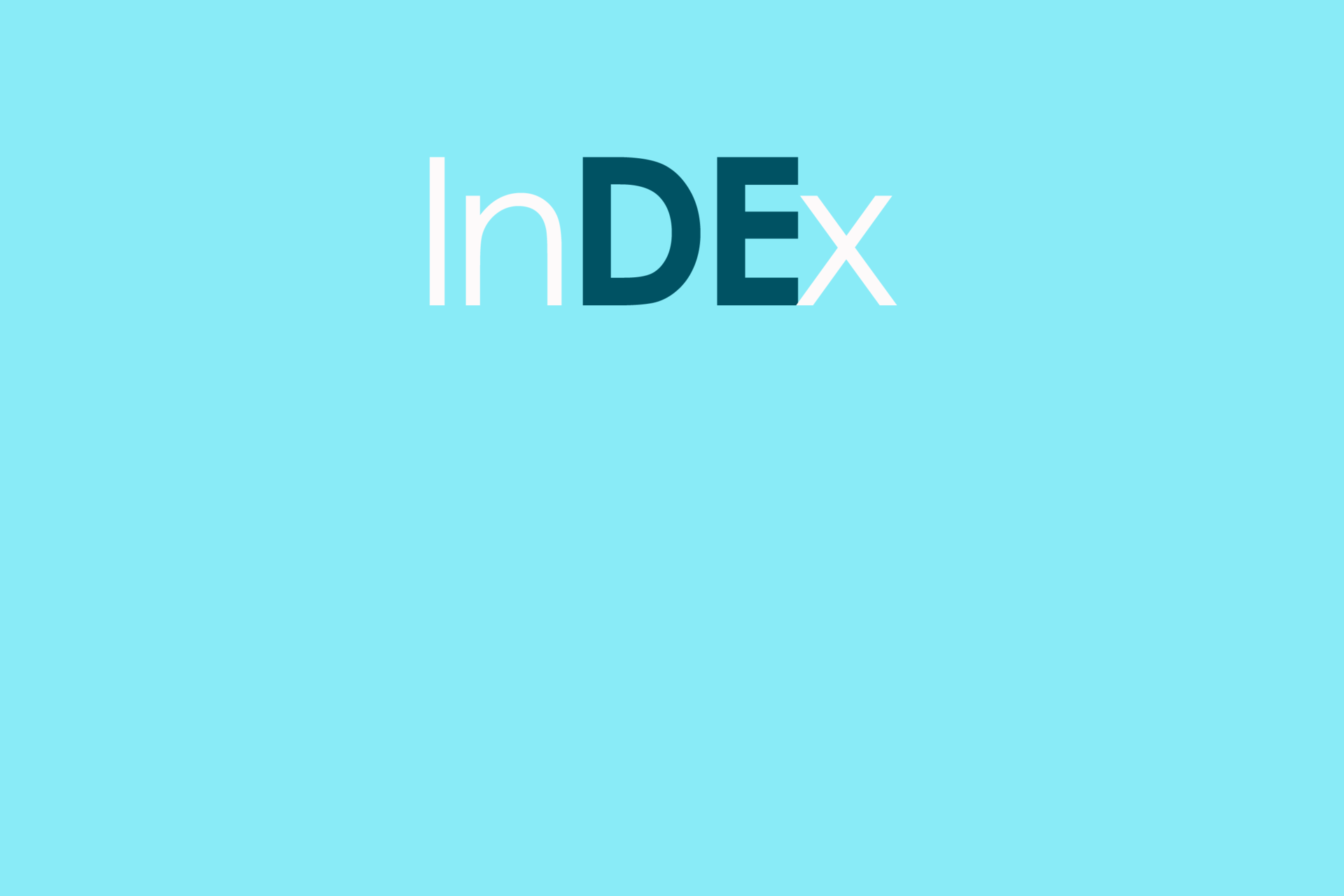

Supplement: Source Code [file NIHMS77548-supplement-Source_Code.zip › resources/android/splash/drawable-land-xxxhdpi-screen.png]

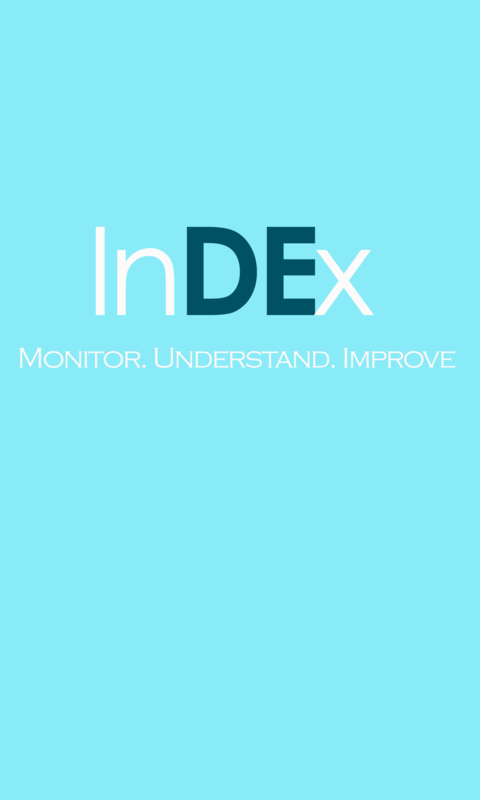

Supplement: Source Code [file NIHMS77548-supplement-Source_Code.zip › resources/android/splash/drawable-port-hdpi-screen.png]

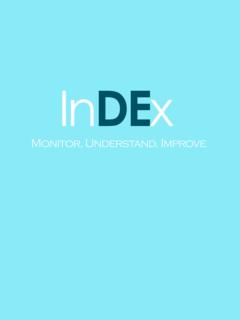

Supplement: Source Code [file NIHMS77548-supplement-Source_Code.zip › resources/android/splash/drawable-port-ldpi-screen.png]

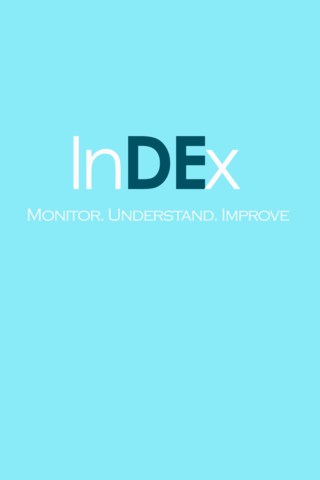

Supplement: Source Code [file NIHMS77548-supplement-Source_Code.zip › resources/android/splash/drawable-port-mdpi-screen.png]

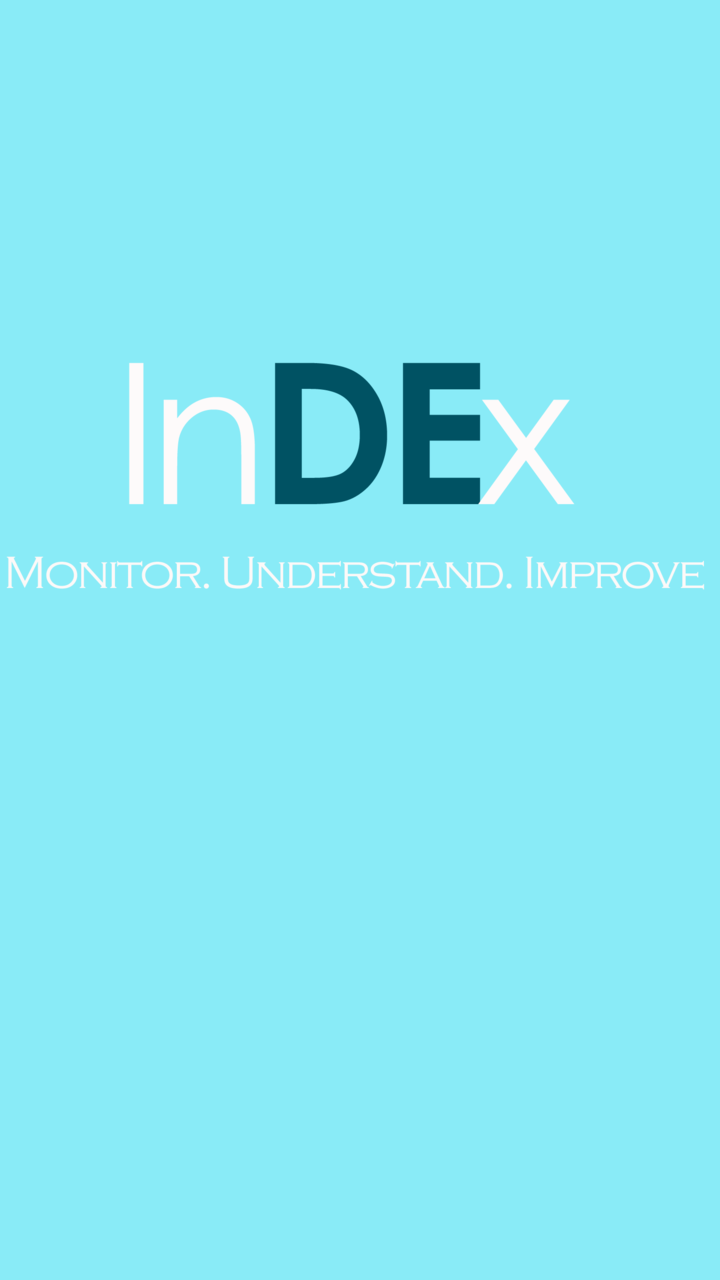

Supplement: Source Code [file NIHMS77548-supplement-Source_Code.zip › resources/android/splash/drawable-port-xhdpi-screen.png]

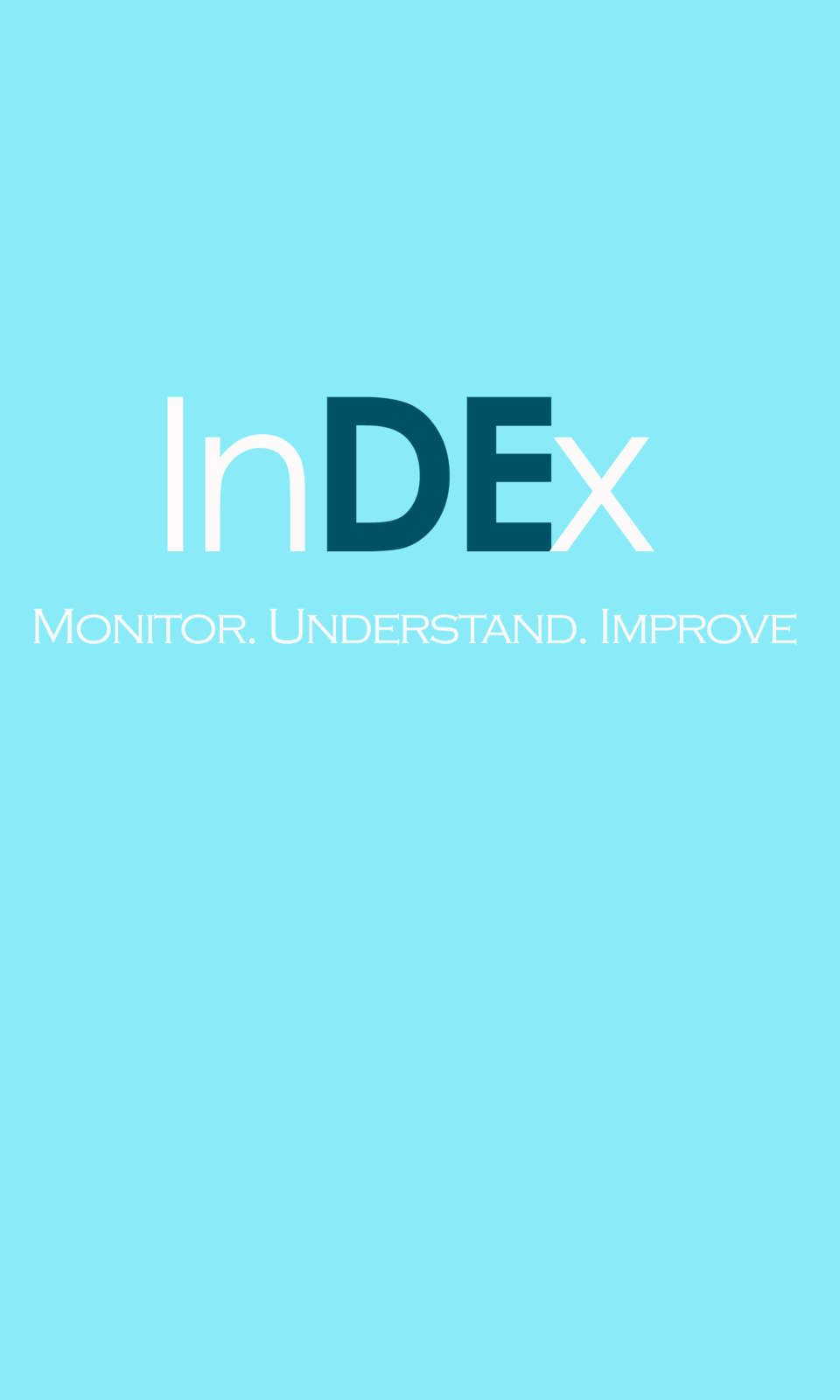

Supplement: Source Code [file NIHMS77548-supplement-Source_Code.zip › resources/android/splash/drawable-port-xxhdpi-screen.png]

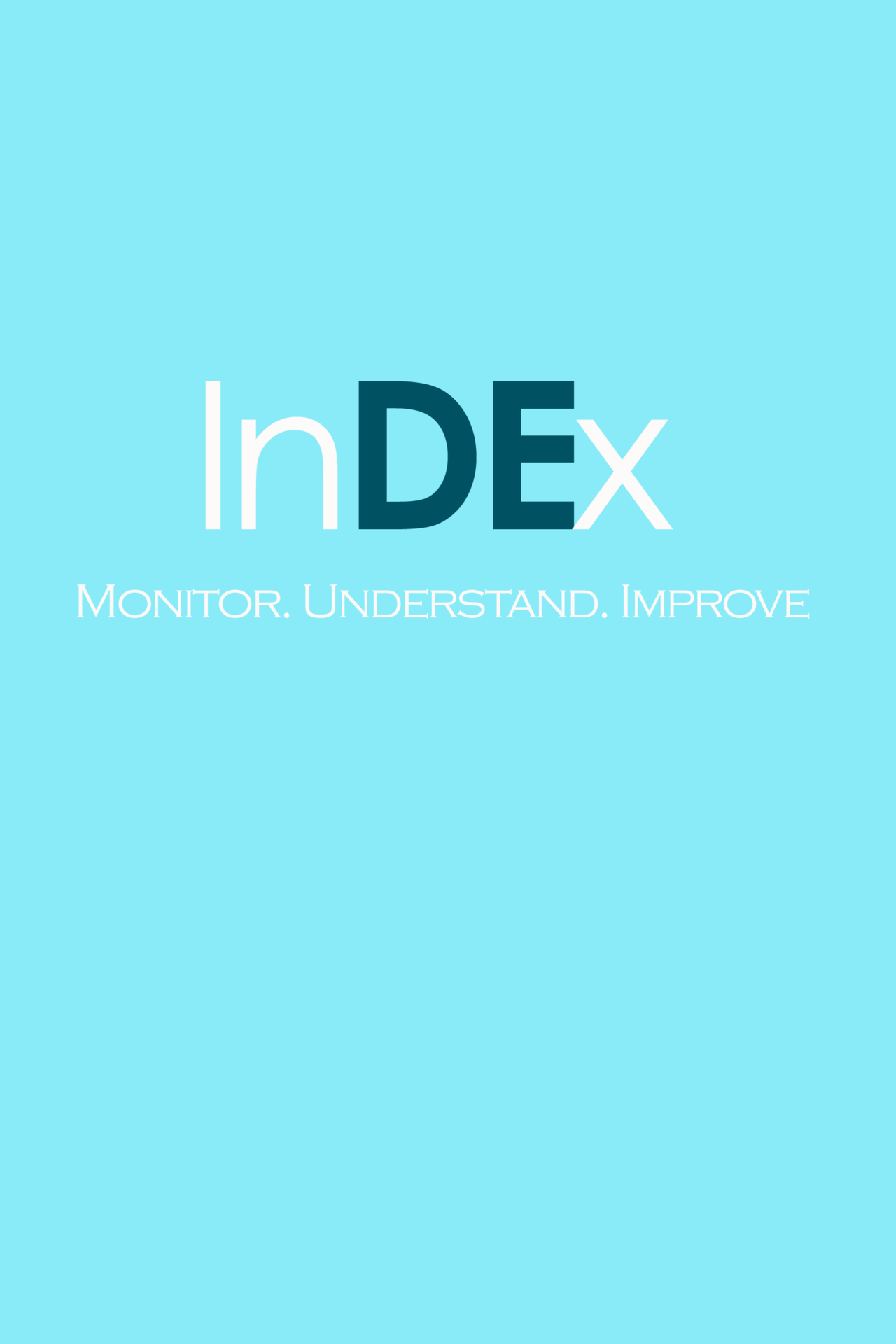

Supplement: Source Code [file NIHMS77548-supplement-Source_Code.zip › resources/android/splash/drawable-port-xxxhdpi-screen.png]

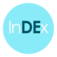

Supplement: Source Code [file NIHMS77548-supplement-Source_Code.zip › resources/ios/icon/icon.png]

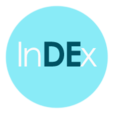

Supplement: Source Code [file NIHMS77548-supplement-Source_Code.zip › resources/ios/icon/icon@2x.png]

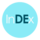

Supplement: Source Code [file NIHMS77548-supplement-Source_Code.zip › resources/ios/icon/icon-40.png]

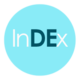

Supplement: Source Code [file NIHMS77548-supplement-Source_Code.zip › resources/ios/icon/icon-40@2x.png]

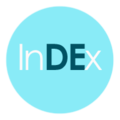

Supplement: Source Code [file NIHMS77548-supplement-Source_Code.zip › resources/ios/icon/icon-40@3x.png]

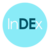

Supplement: Source Code [file NIHMS77548-supplement-Source_Code.zip › resources/ios/icon/icon-50.png]

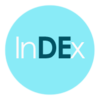

Supplement: Source Code [file NIHMS77548-supplement-Source_Code.zip › resources/ios/icon/icon-50@2x.png]

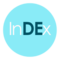

Supplement: Source Code [file NIHMS77548-supplement-Source_Code.zip › resources/ios/icon/icon-60.png]

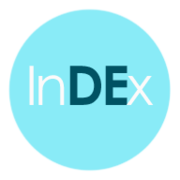

Supplement: Source Code [file NIHMS77548-supplement-Source_Code.zip › resources/ios/icon/icon-60@3x.png]

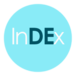

Supplement: Source Code [file NIHMS77548-supplement-Source_Code.zip › resources/ios/icon/icon-76.png]

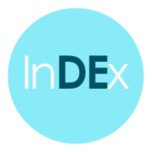

Supplement: Source Code [file NIHMS77548-supplement-Source_Code.zip › resources/ios/icon/icon-76@2x.png]

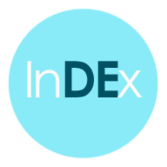

Supplement: Source Code [file NIHMS77548-supplement-Source_Code.zip › resources/ios/icon/icon-83.5@2x.png]

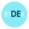

Supplement: Source Code [file NIHMS77548-supplement-Source_Code.zip › resources/ios/icon/icon-small.png]

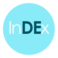

Supplement: Source Code [file NIHMS77548-supplement-Source_Code.zip › resources/ios/icon/icon-small@2x.png]

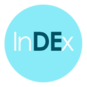

Supplement: Source Code [file NIHMS77548-supplement-Source_Code.zip › resources/ios/icon/icon-small@3x.png]

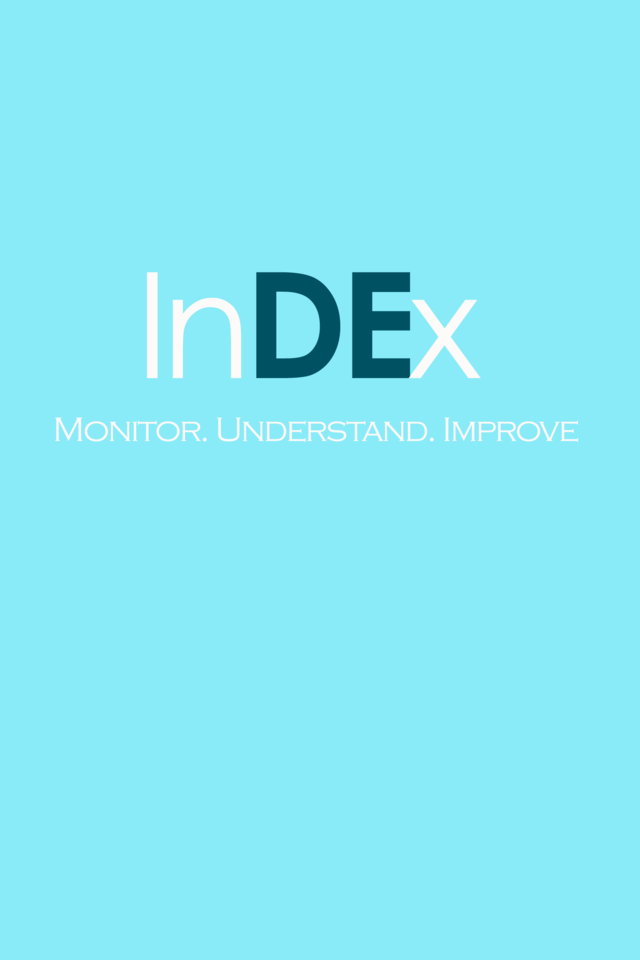

Supplement: Source Code [file NIHMS77548-supplement-Source_Code.zip › resources/ios/splash/Default@2x~iphone.png]

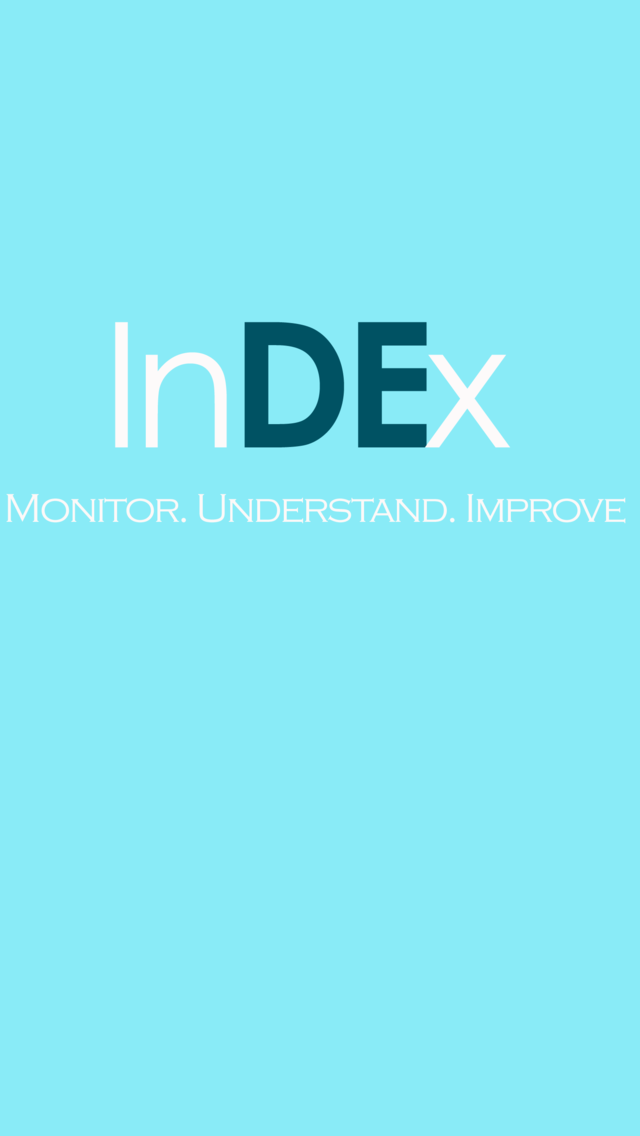

Supplement: Source Code [file NIHMS77548-supplement-Source_Code.zip › resources/ios/splash/Default-568h@2x~iphone.png]

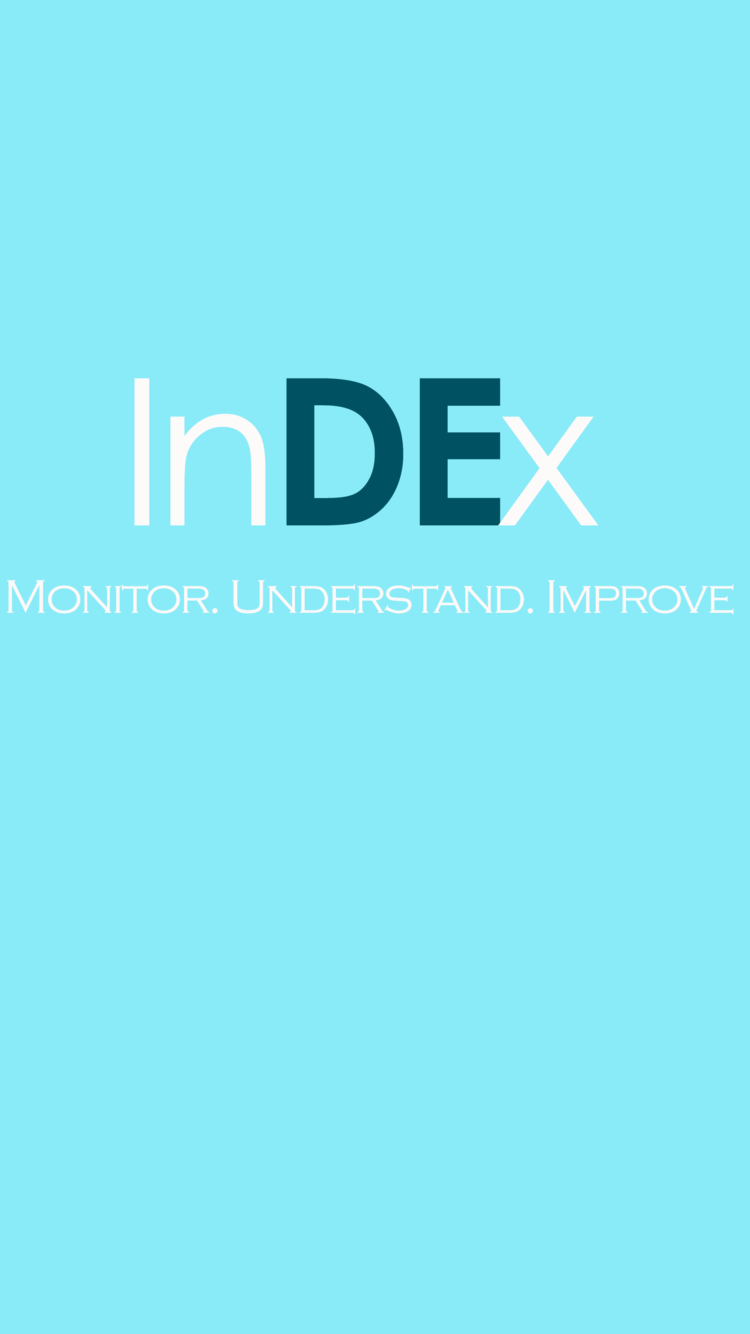

Supplement: Source Code [file NIHMS77548-supplement-Source_Code.zip › resources/ios/splash/Default-667h.png]

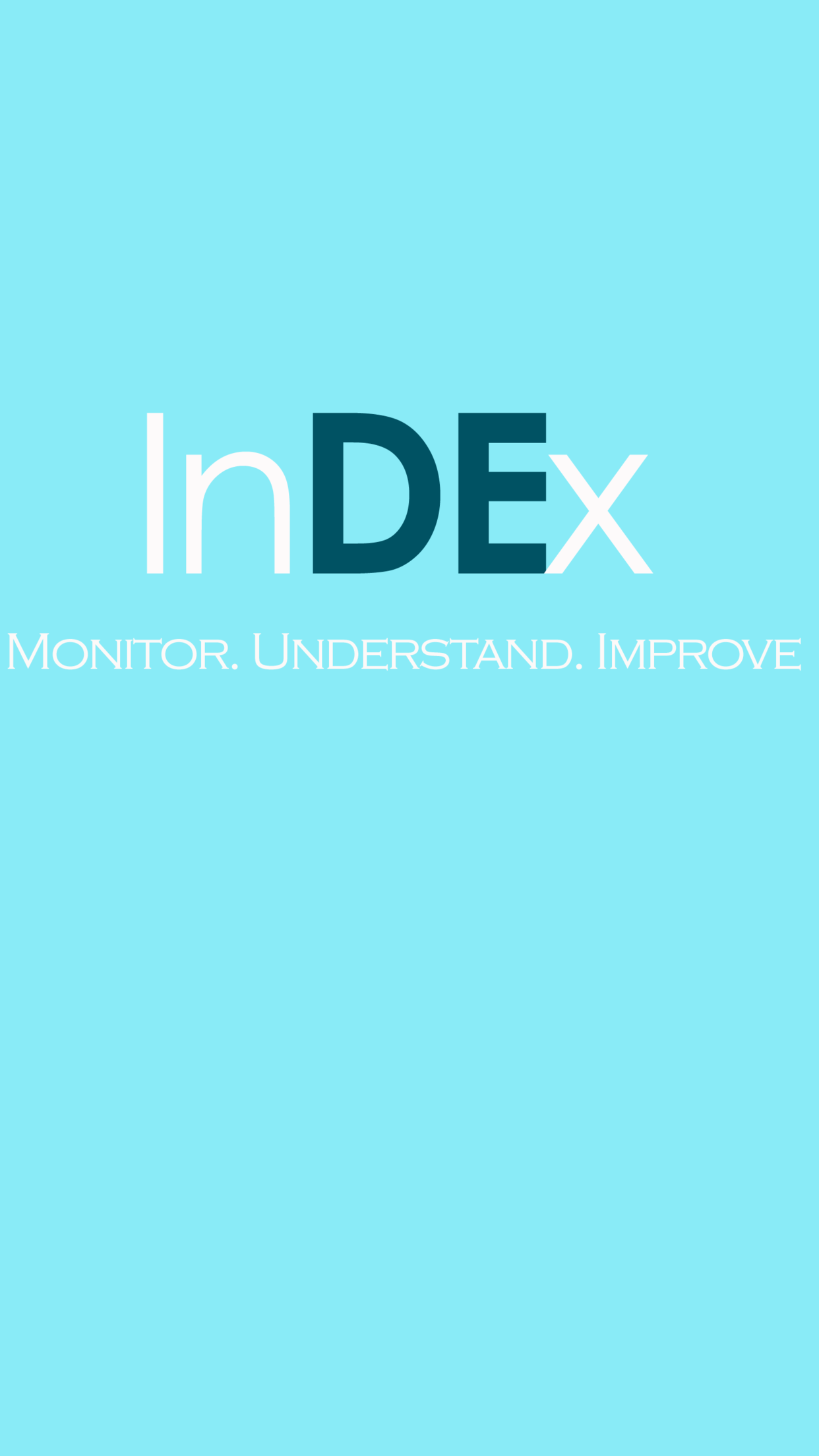

Supplement: Source Code [file NIHMS77548-supplement-Source_Code.zip › resources/ios/splash/Default-736h.png]

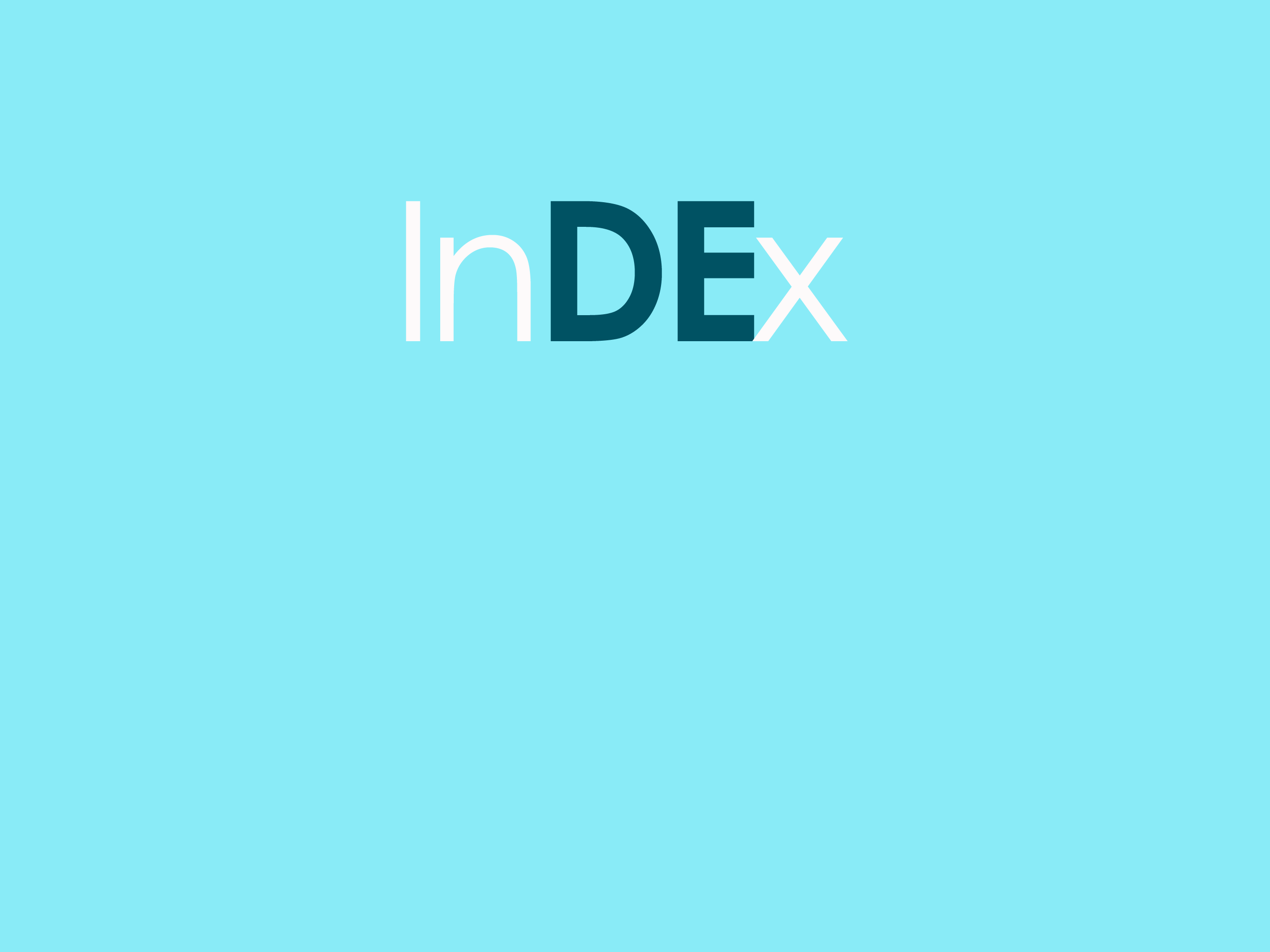

Supplement: Source Code [file NIHMS77548-supplement-Source_Code.zip › resources/ios/splash/Default-Landscape@~ipadpro.png]

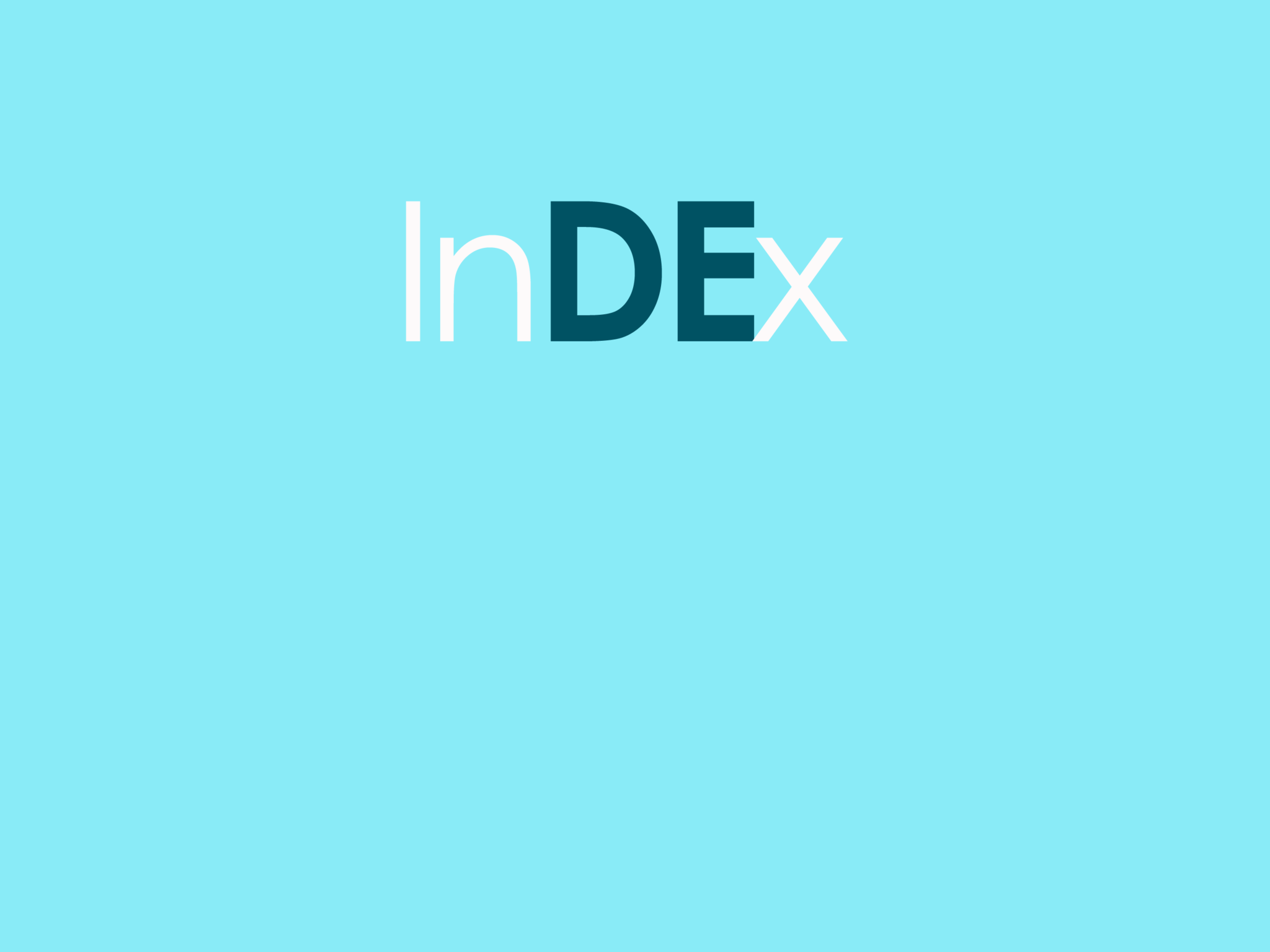

Supplement: Source Code [file NIHMS77548-supplement-Source_Code.zip › resources/ios/splash/Default-Landscape@2x~ipad.png]

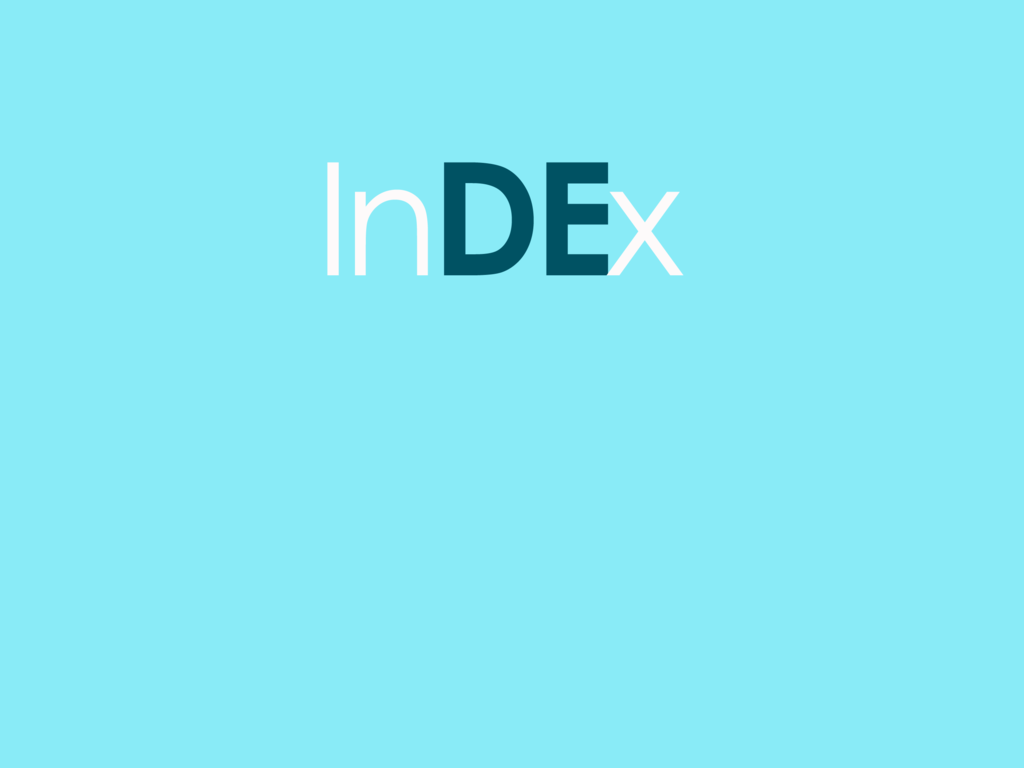

Supplement: Source Code [file NIHMS77548-supplement-Source_Code.zip › resources/ios/splash/Default-Landscape~ipad.png]

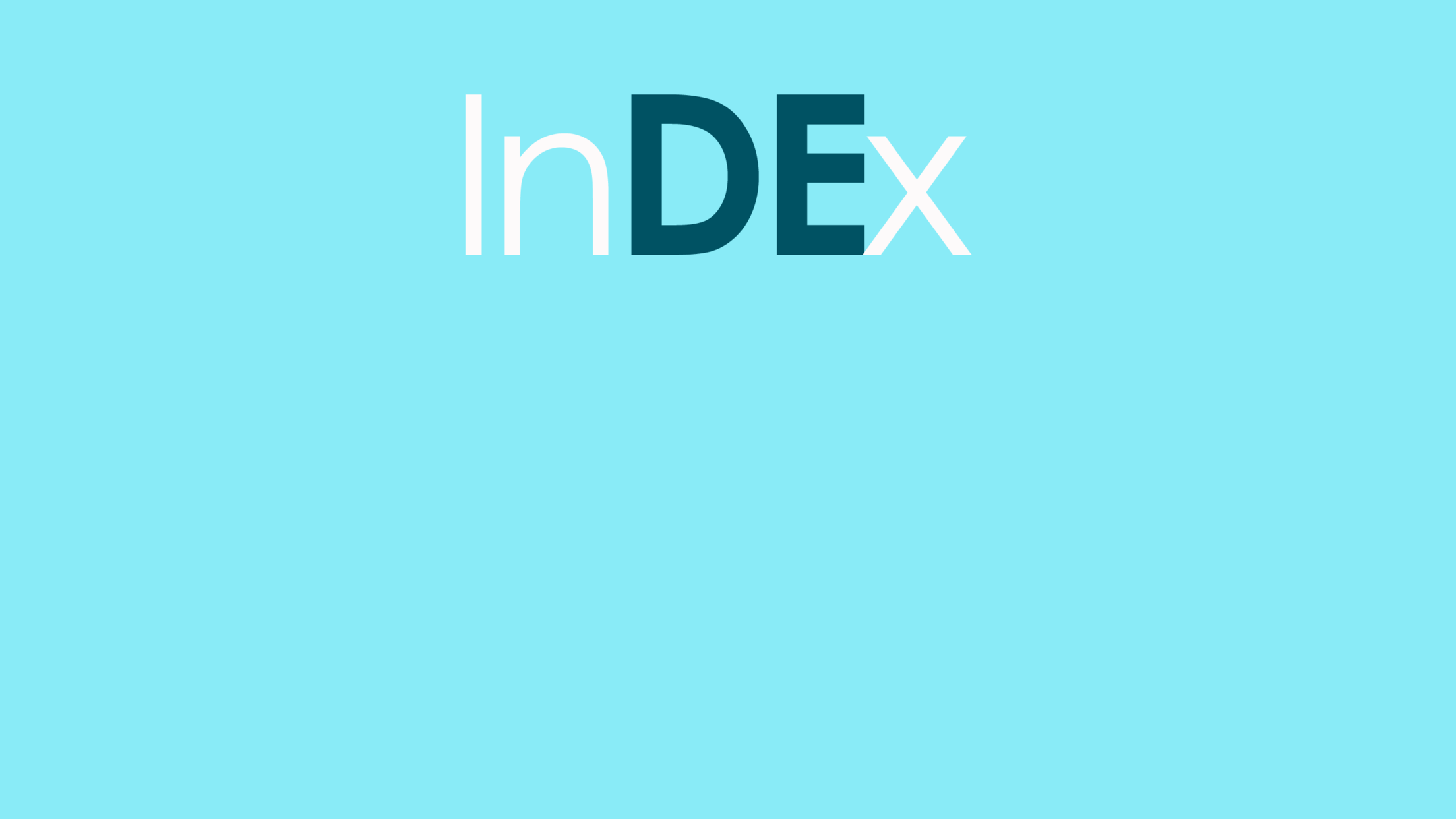

Supplement: Source Code [file NIHMS77548-supplement-Source_Code.zip › resources/ios/splash/Default-Landscape-736h.png]

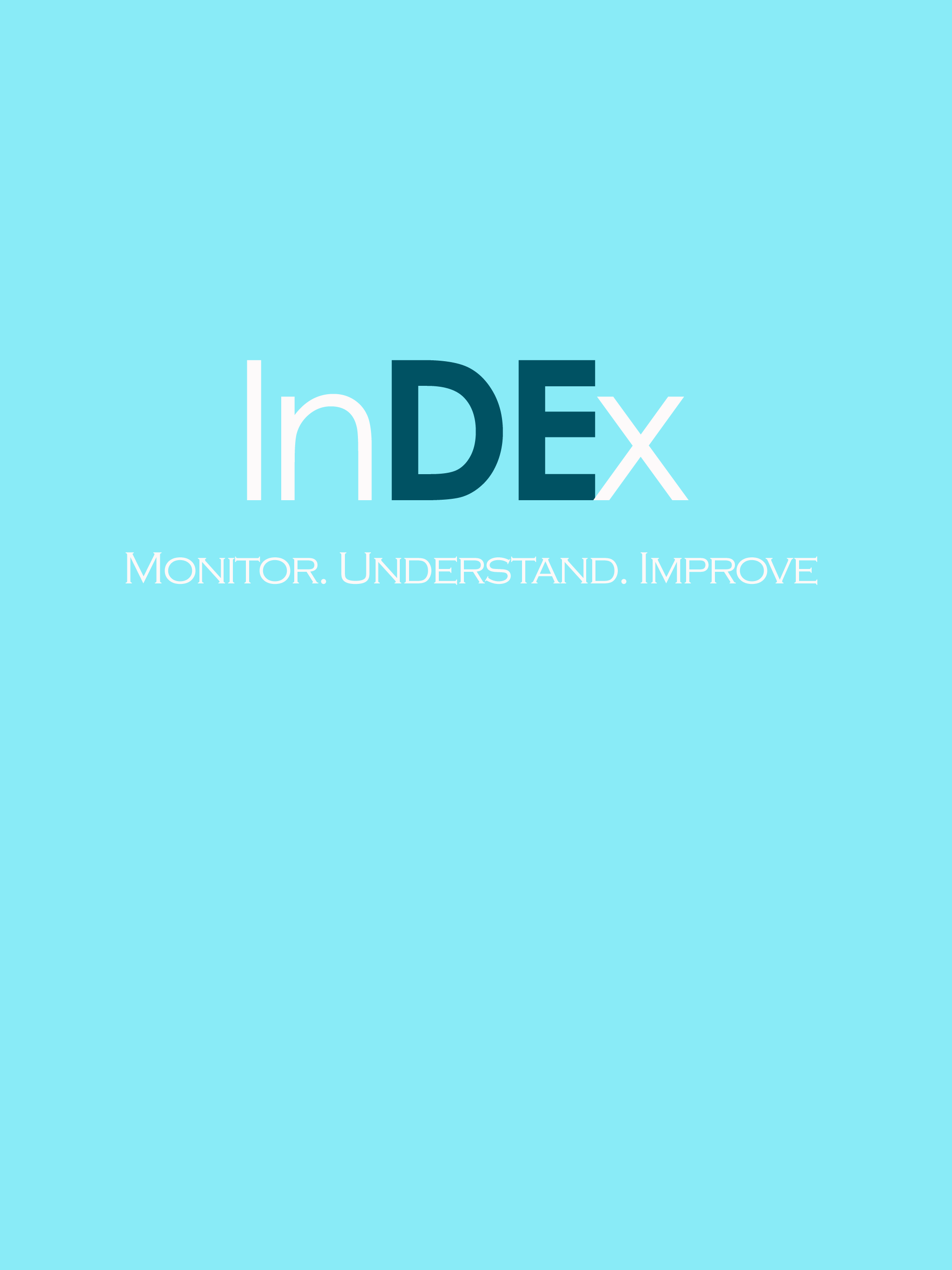

Supplement: Source Code [file NIHMS77548-supplement-Source_Code.zip › resources/ios/splash/Default-Portrait@~ipadpro.png]

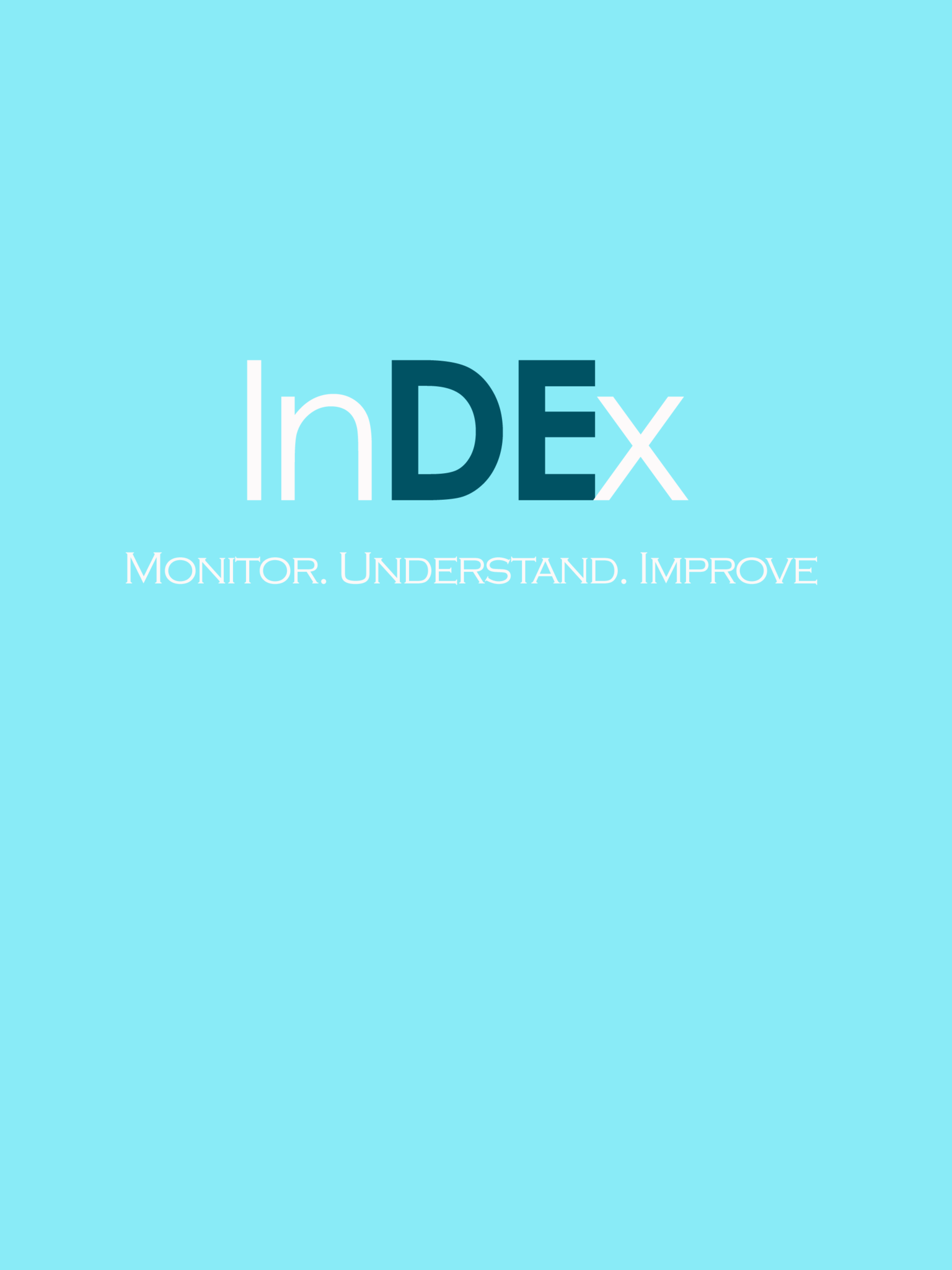

Supplement: Source Code [file NIHMS77548-supplement-Source_Code.zip › resources/ios/splash/Default-Portrait@2x~ipad.png]

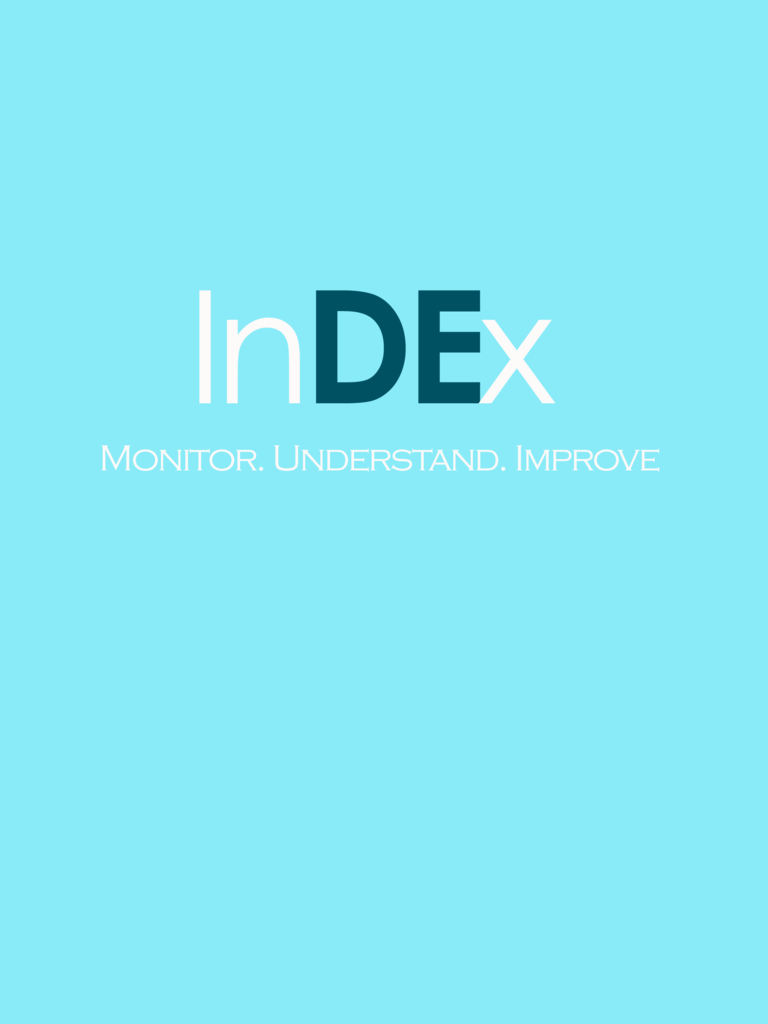

Supplement: Source Code [file NIHMS77548-supplement-Source_Code.zip › resources/ios/splash/Default-Portrait~ipad.png]

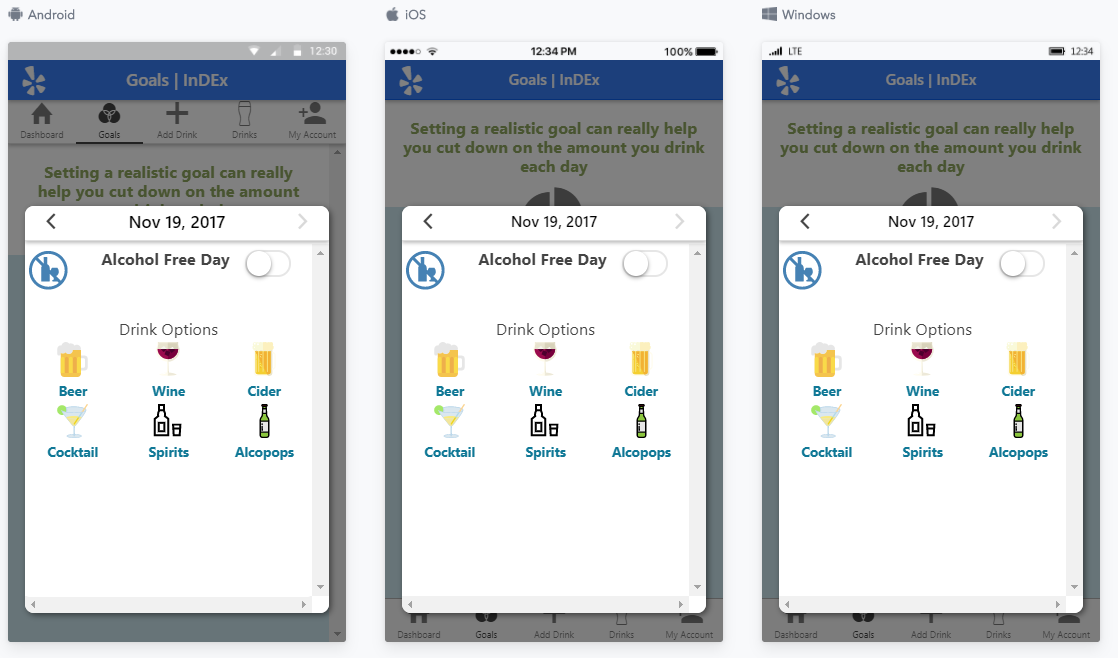

Supplement: Source Code [file NIHMS77548-supplement-Source_Code.zip › resources/screenshots/add_drink.png]

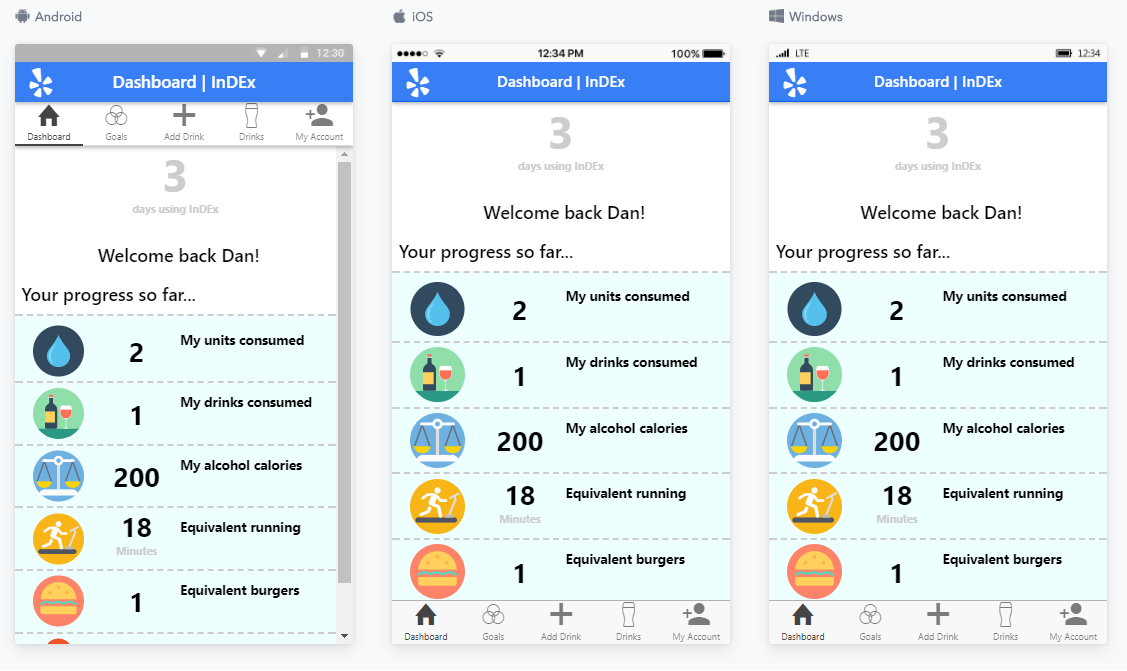

Supplement: Source Code [file NIHMS77548-supplement-Source_Code.zip › resources/screenshots/dashboard.png]

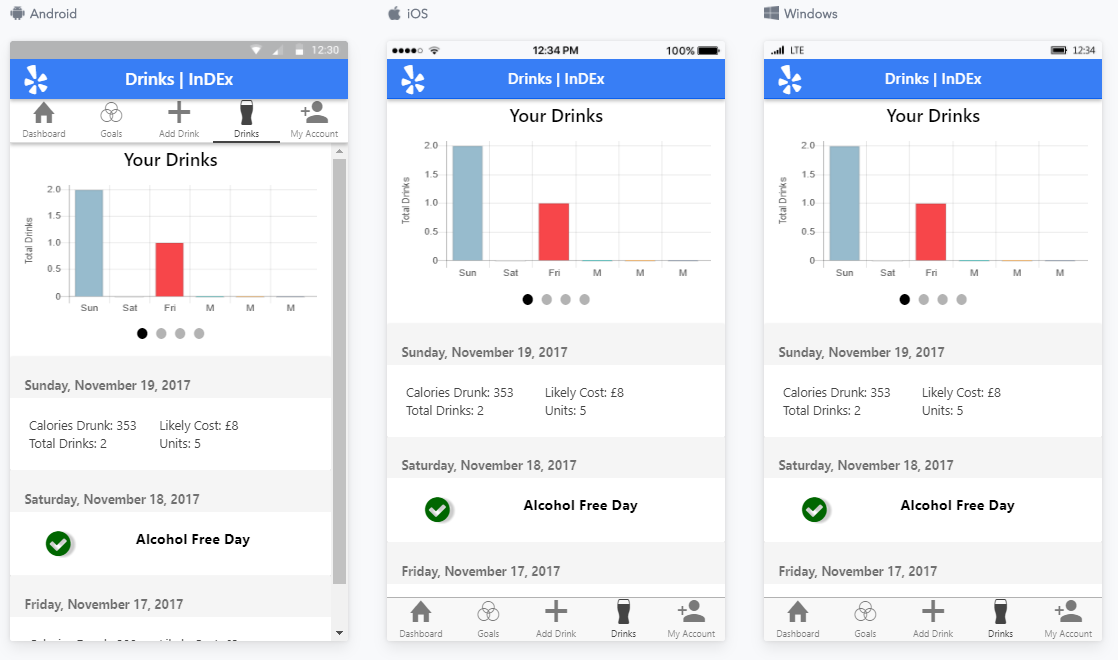

Supplement: Source Code [file NIHMS77548-supplement-Source_Code.zip › resources/screenshots/drink_diary.png]

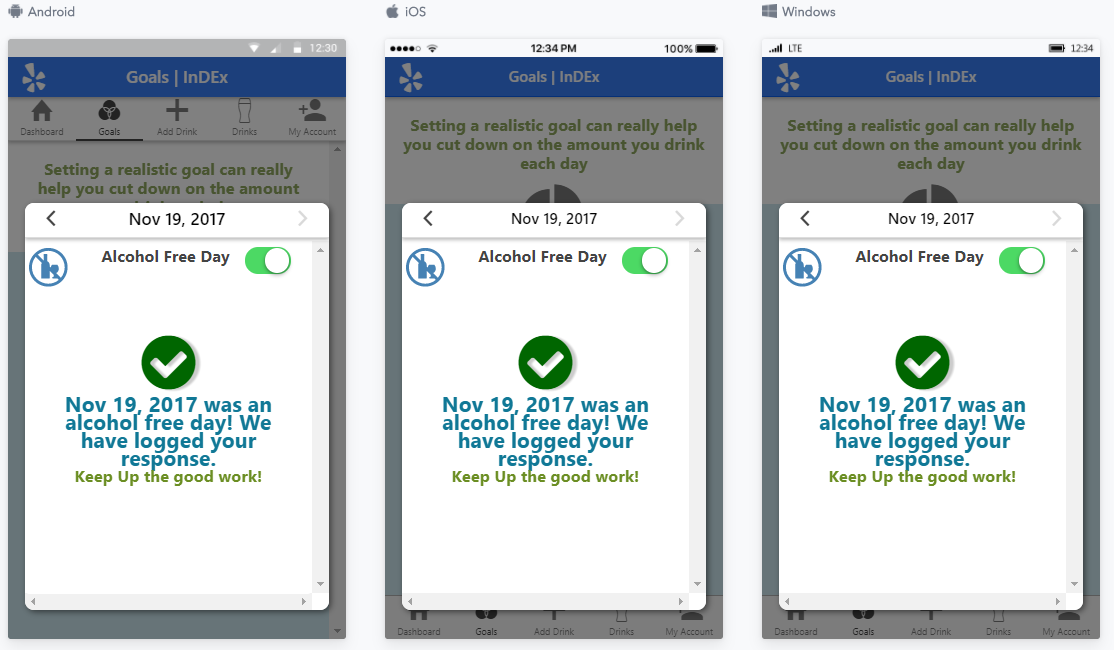

Supplement: Source Code [file NIHMS77548-supplement-Source_Code.zip › resources/screenshots/drink_free_day.png]

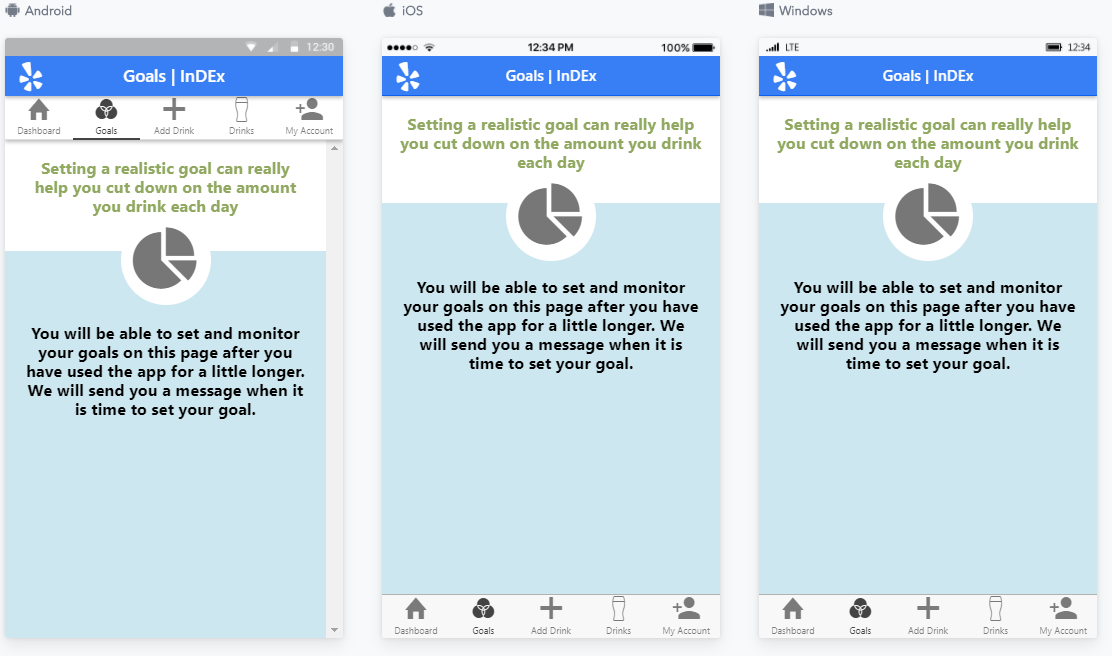

Supplement: Source Code [file NIHMS77548-supplement-Source_Code.zip › resources/screenshots/goals_first_week.png]

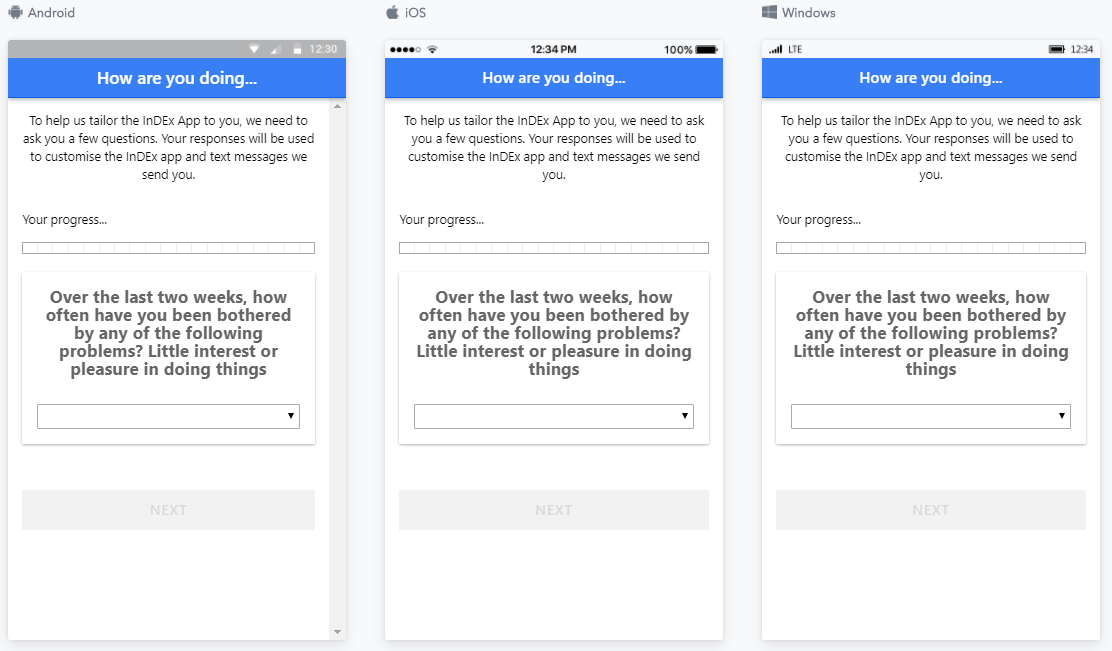

Supplement: Source Code [file NIHMS77548-supplement-Source_Code.zip › resources/screenshots/screening.png]

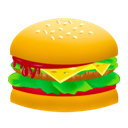

Supplement: Source Code [file NIHMS77548-supplement-Source_Code.zip › www/img/burger_icon.png]

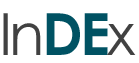

Supplement: Source Code [file NIHMS77548-supplement-Source_Code.zip › www/img/index_logo.png]

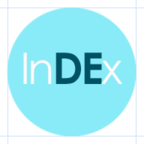

Supplement: Source Code [file NIHMS77548-supplement-Source_Code.zip › www/img/index-icon.png]

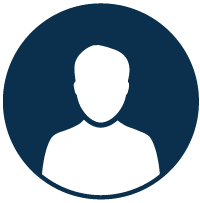

Supplement: Source Code [file NIHMS77548-supplement-Source_Code.zip › www/img/placeholder_image.png]

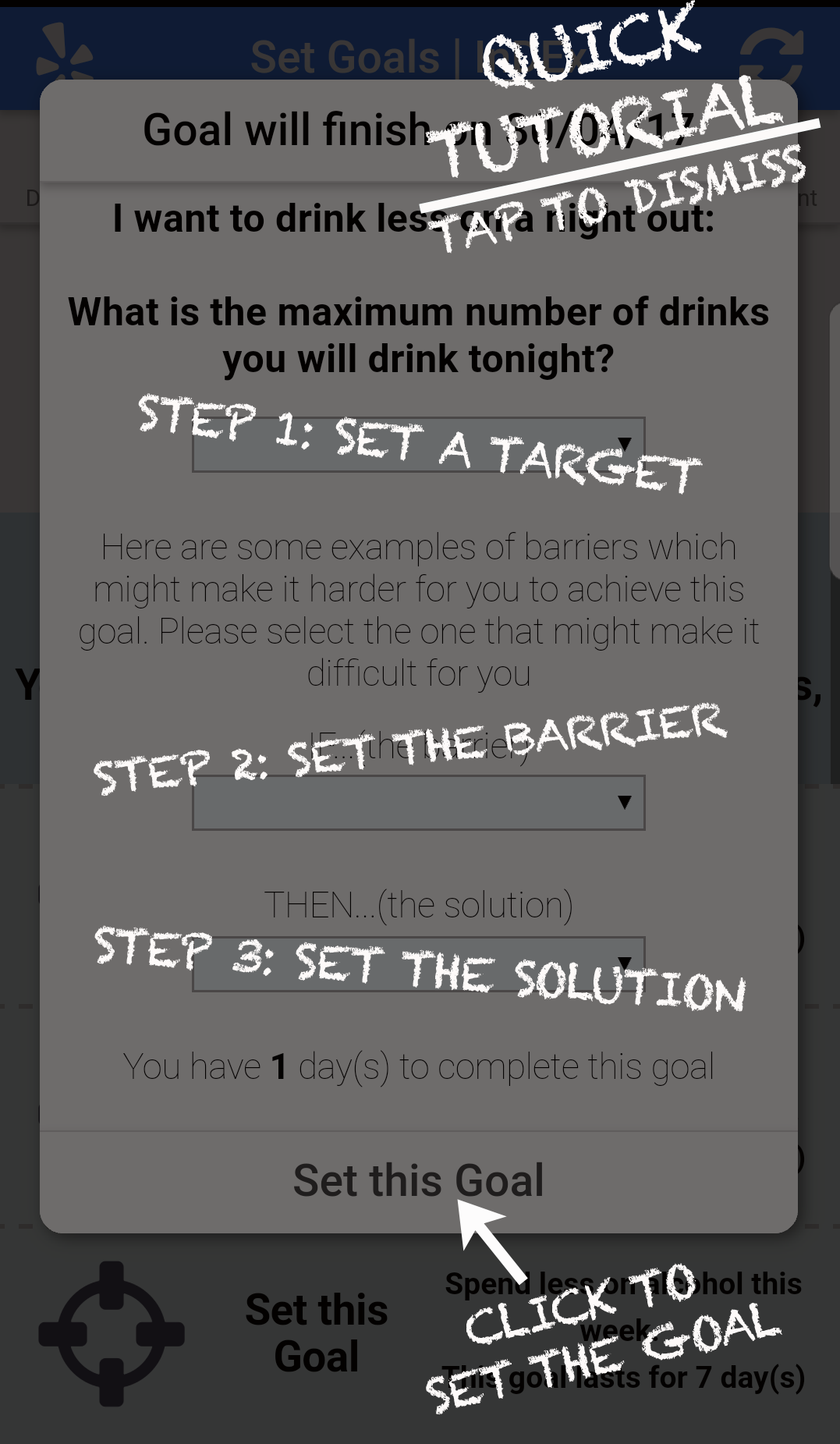

Supplement: Source Code [file NIHMS77548-supplement-Source_Code.zip › www/img/walkthrough/add_goal_stage5.png]

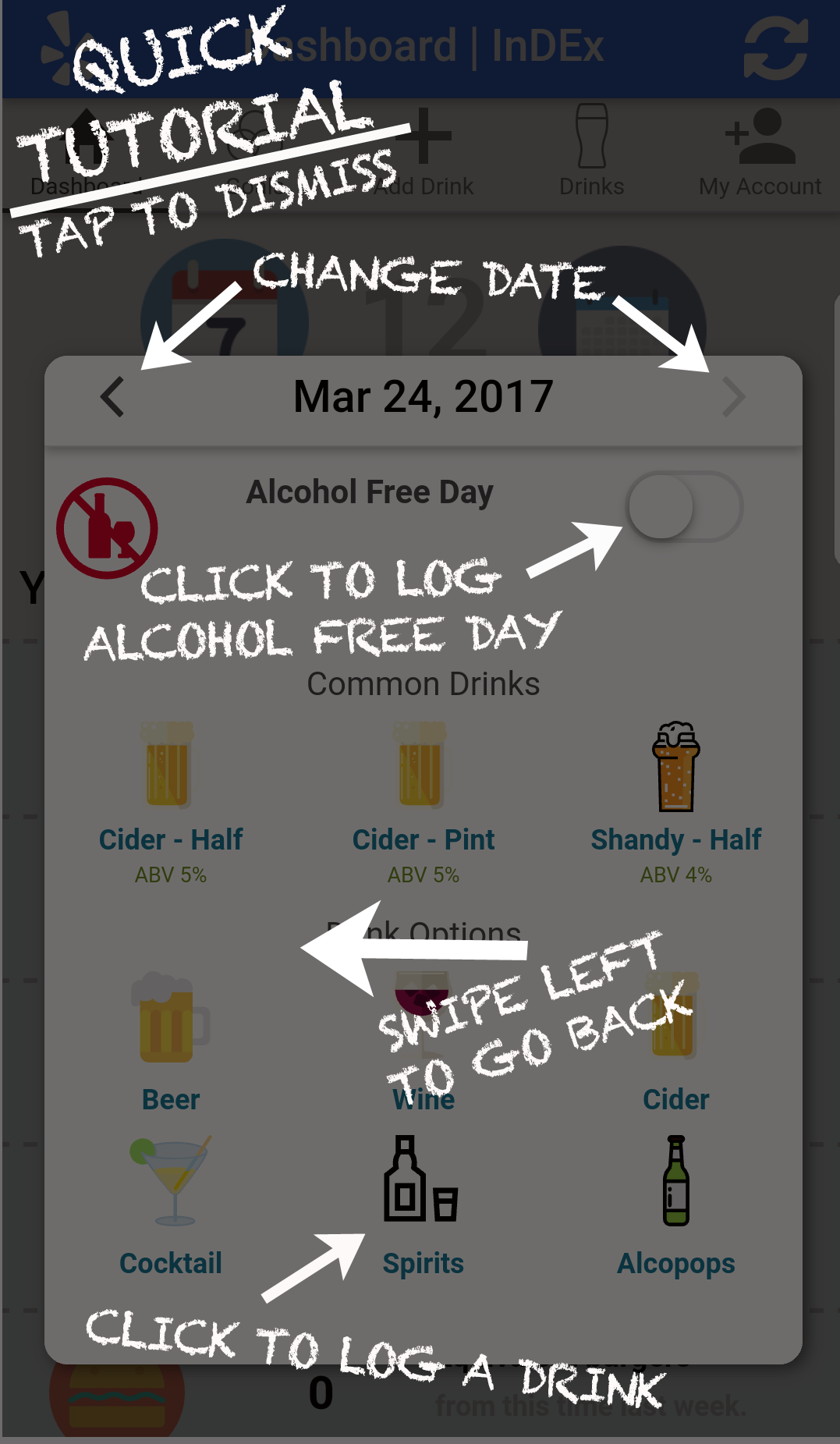

Supplement: Source Code [file NIHMS77548-supplement-Source_Code.zip › www/img/walkthrough/add-drinks-stage2.png]

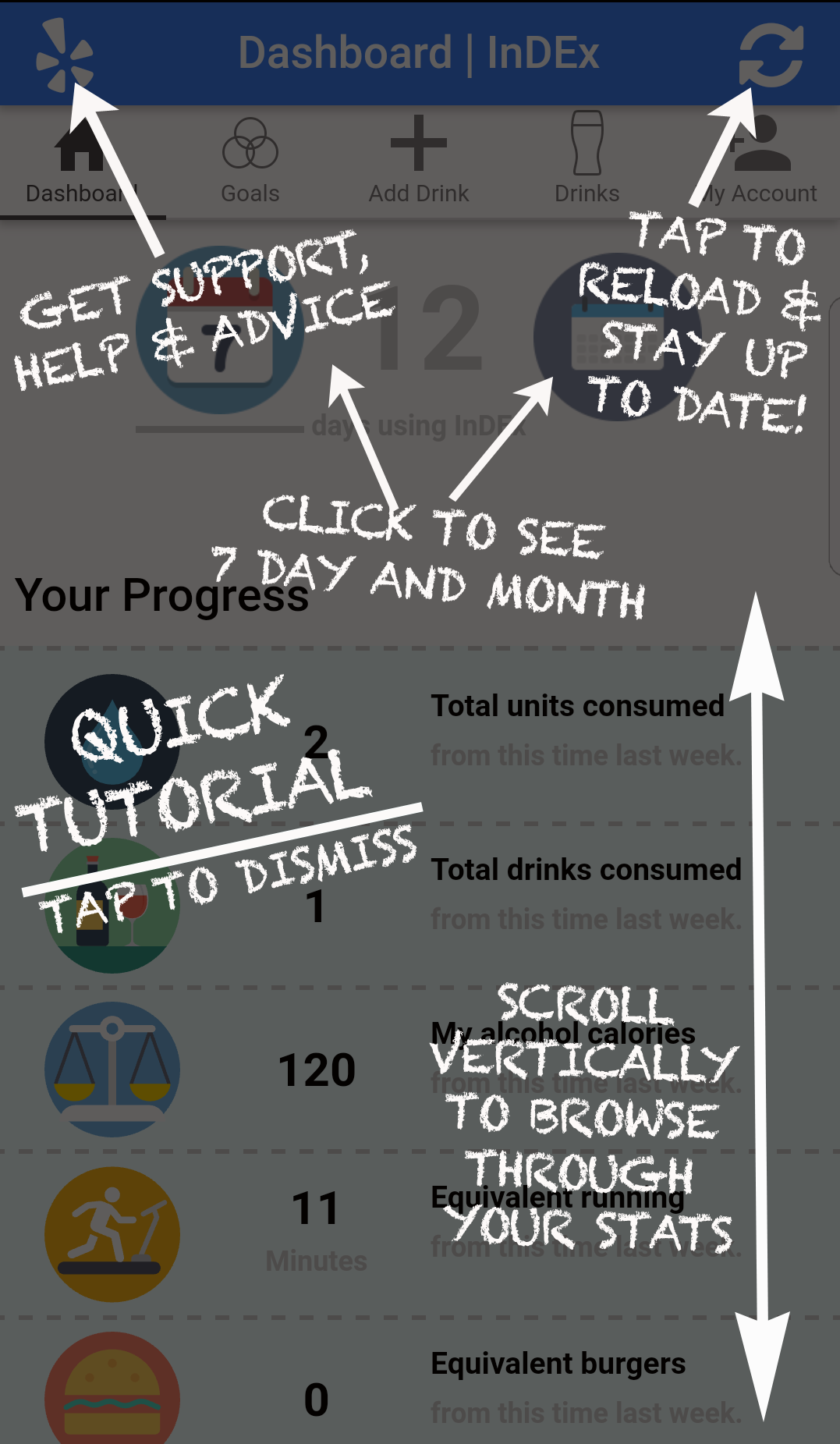

Supplement: Source Code [file NIHMS77548-supplement-Source_Code.zip › www/img/walkthrough/dashboard-stage1.png]

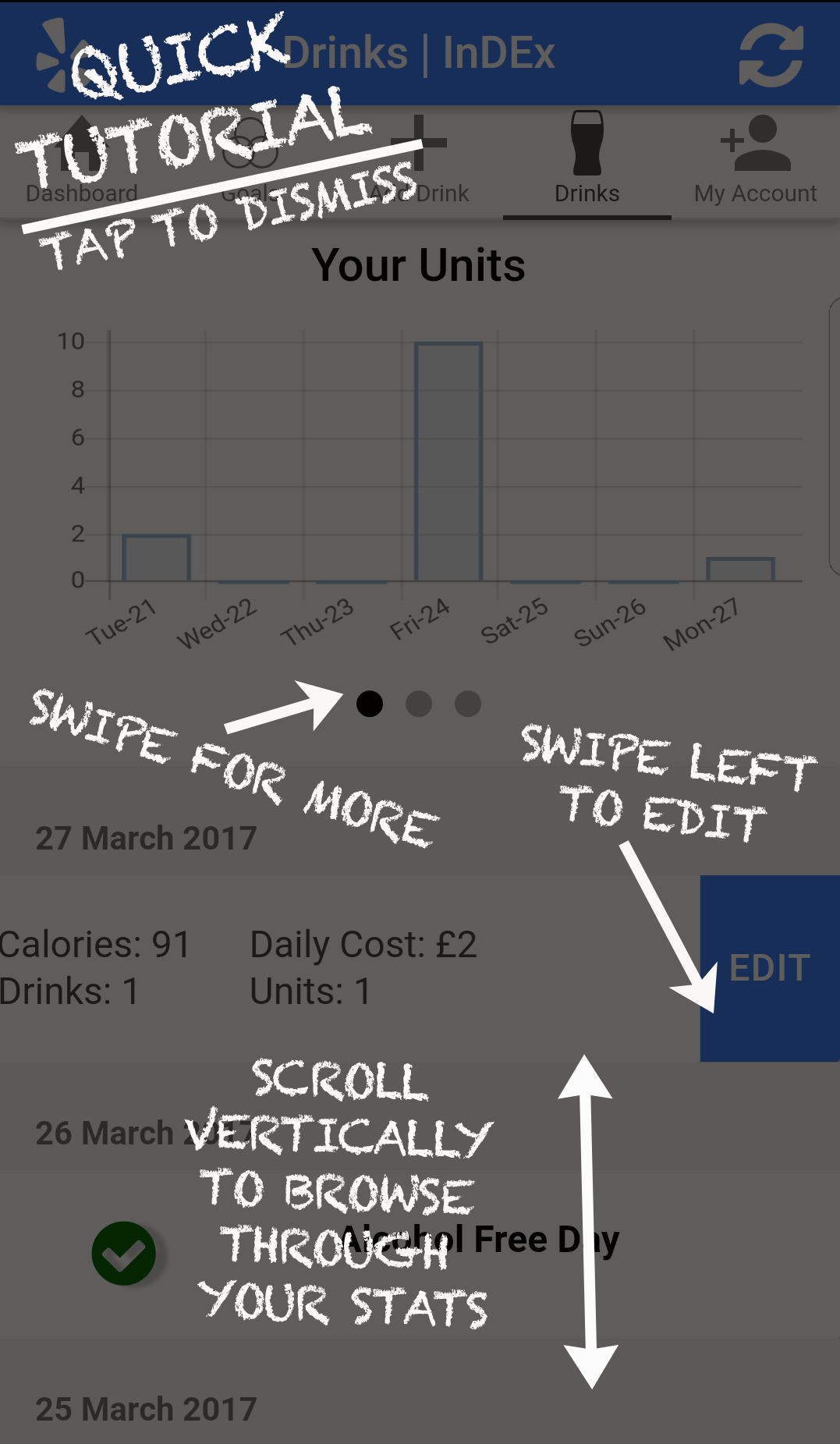

Supplement: Source Code [file NIHMS77548-supplement-Source_Code.zip › www/img/walkthrough/drinkdiary-stage3.png]

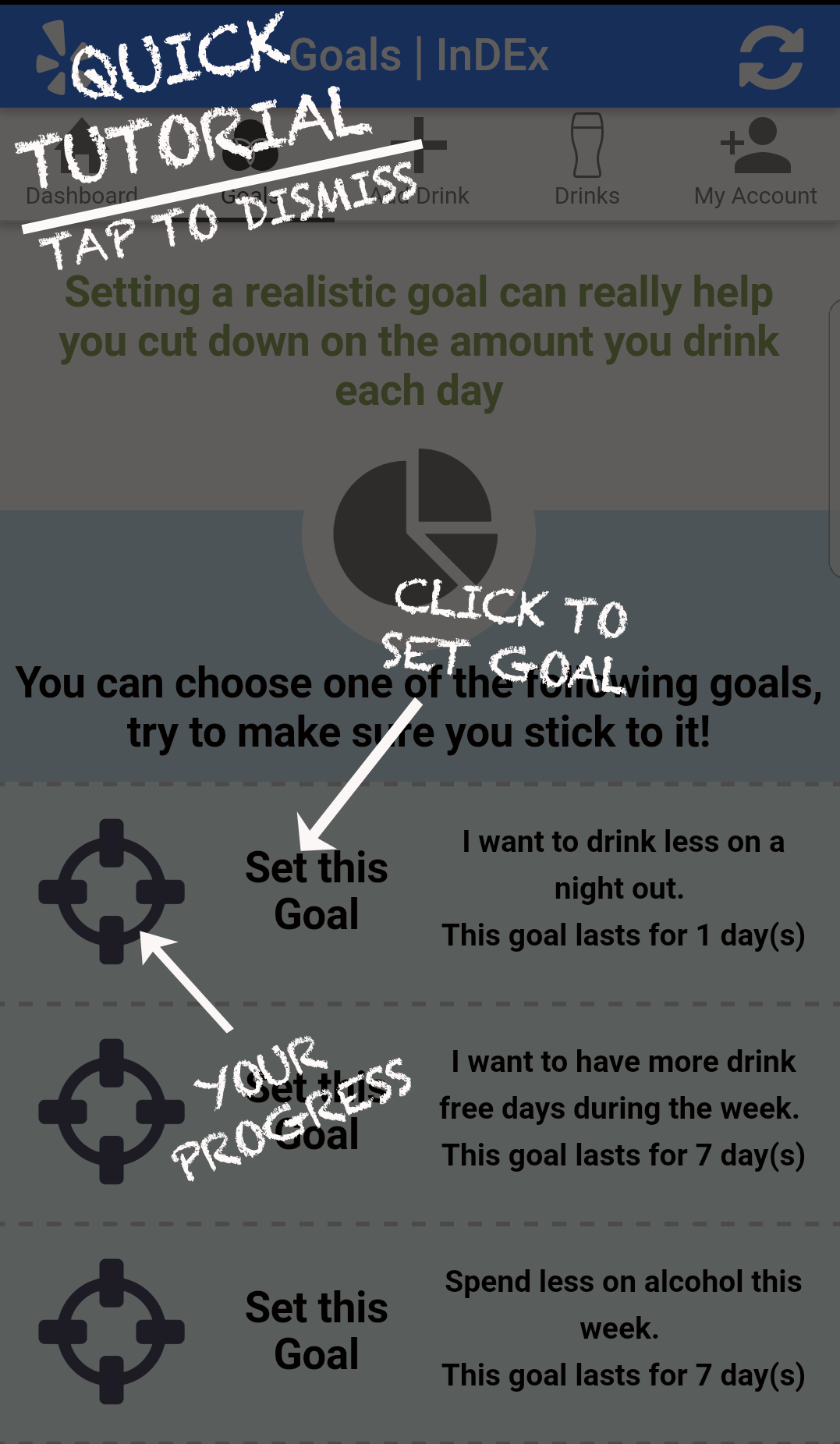

Supplement: Source Code [file NIHMS77548-supplement-Source_Code.zip › www/img/walkthrough/goals_stage4.png]

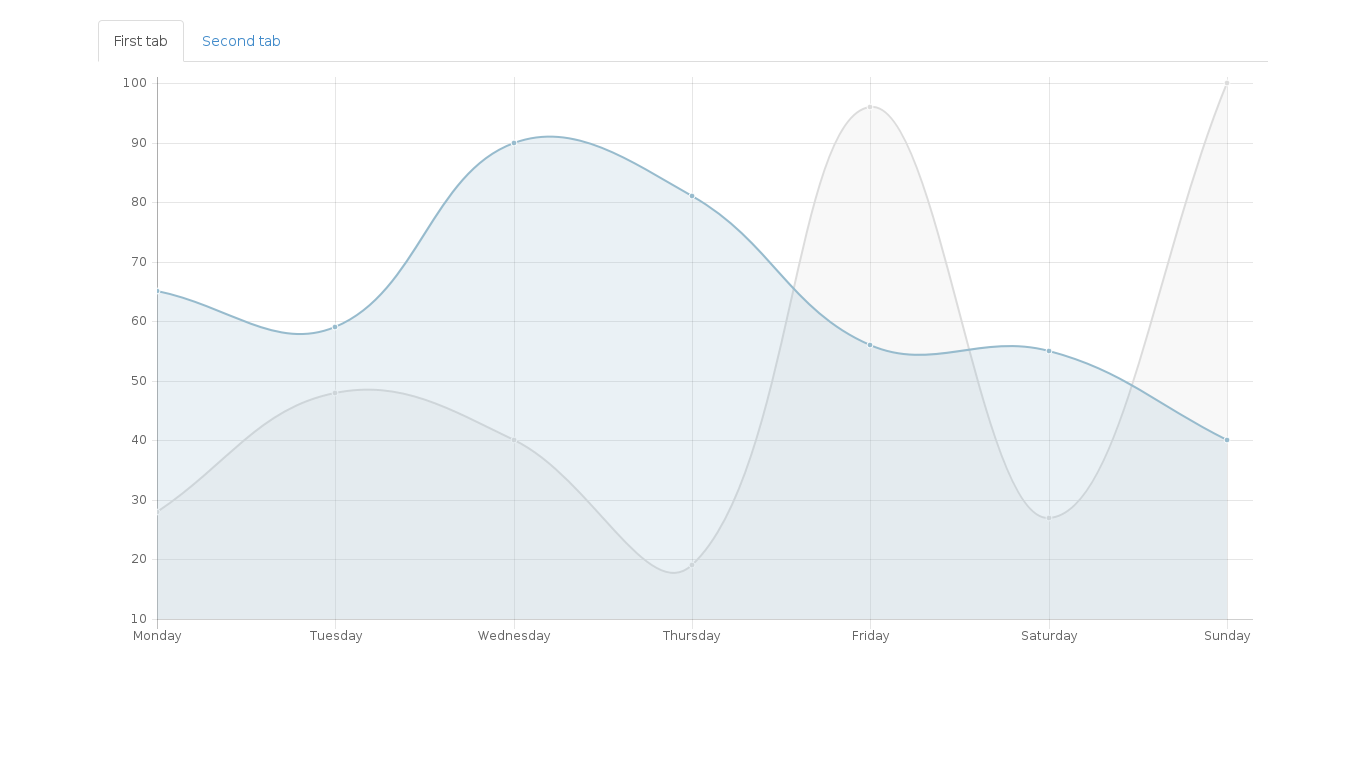

Supplement: Source Code [file NIHMS77548-supplement-Source_Code.zip › www/lib/angular-chart.js/test/fixtures/29-tabs.png]

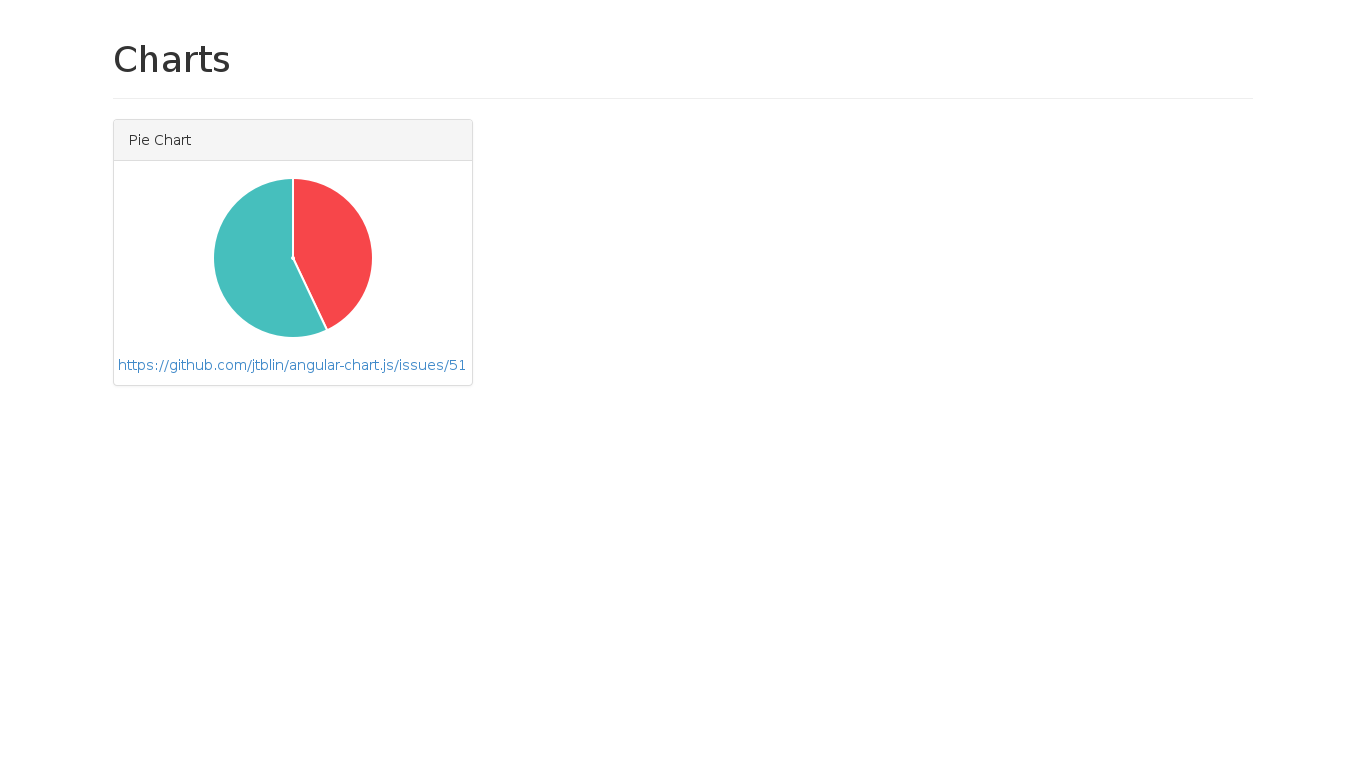

Supplement: Source Code [file NIHMS77548-supplement-Source_Code.zip › www/lib/angular-chart.js/test/fixtures/51-pie-update-colours.png]

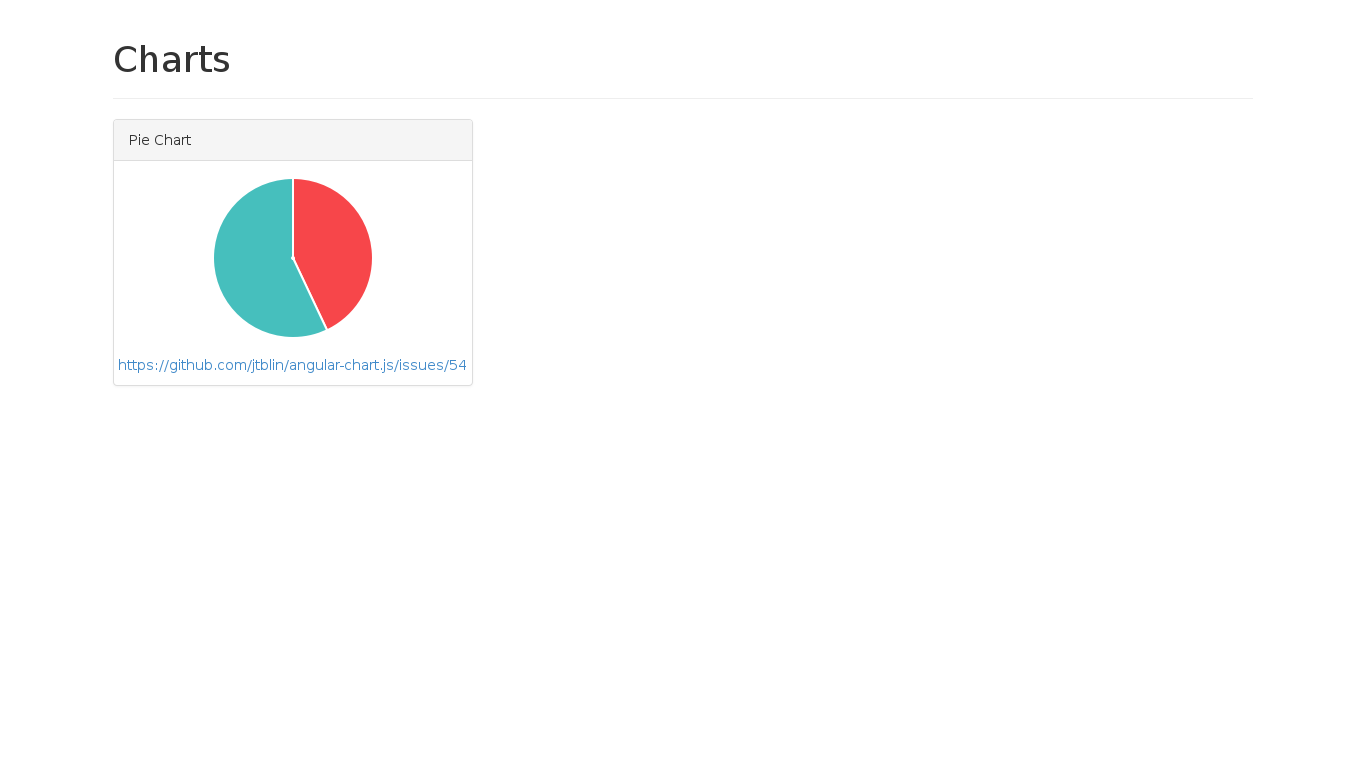

Supplement: Source Code [file NIHMS77548-supplement-Source_Code.zip › www/lib/angular-chart.js/test/fixtures/54-not-enough-colours.png]

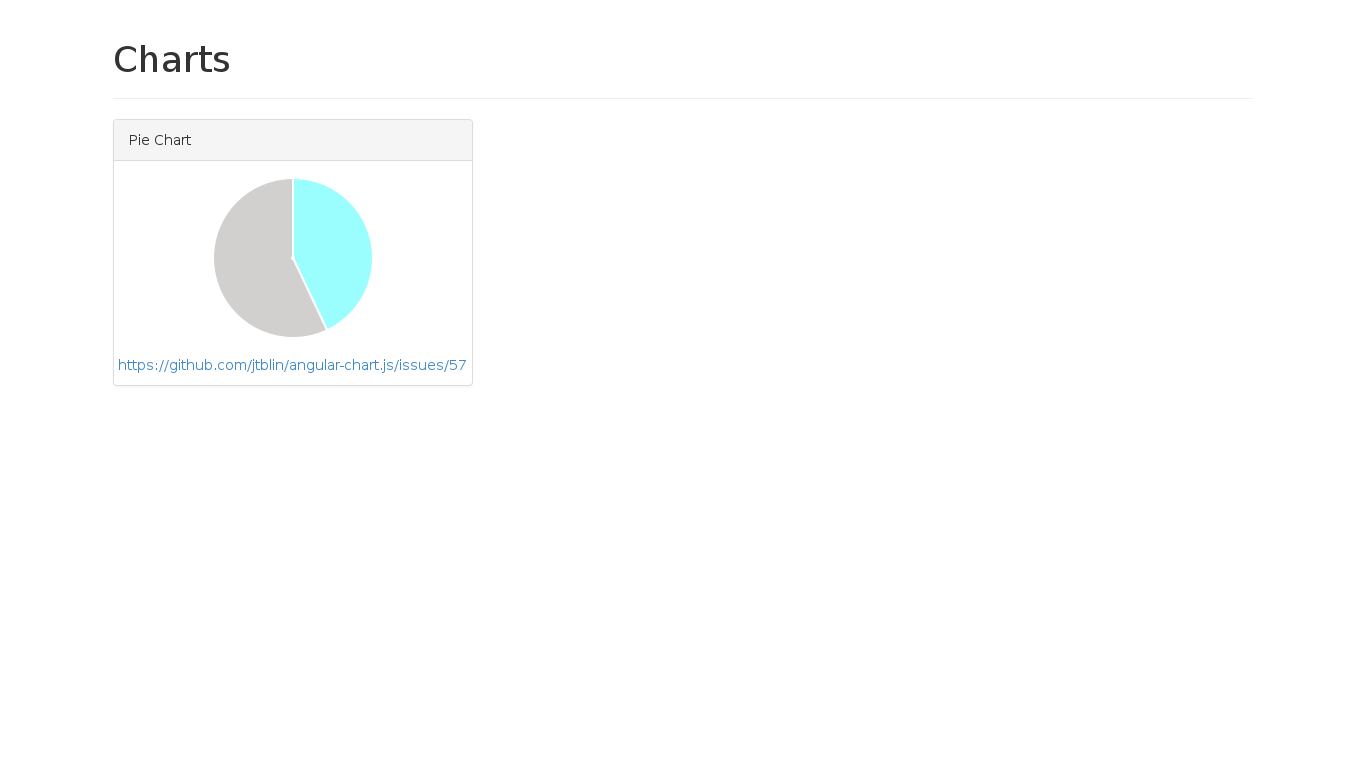

Supplement: Source Code [file NIHMS77548-supplement-Source_Code.zip › www/lib/angular-chart.js/test/fixtures/57-hex-colours.png]

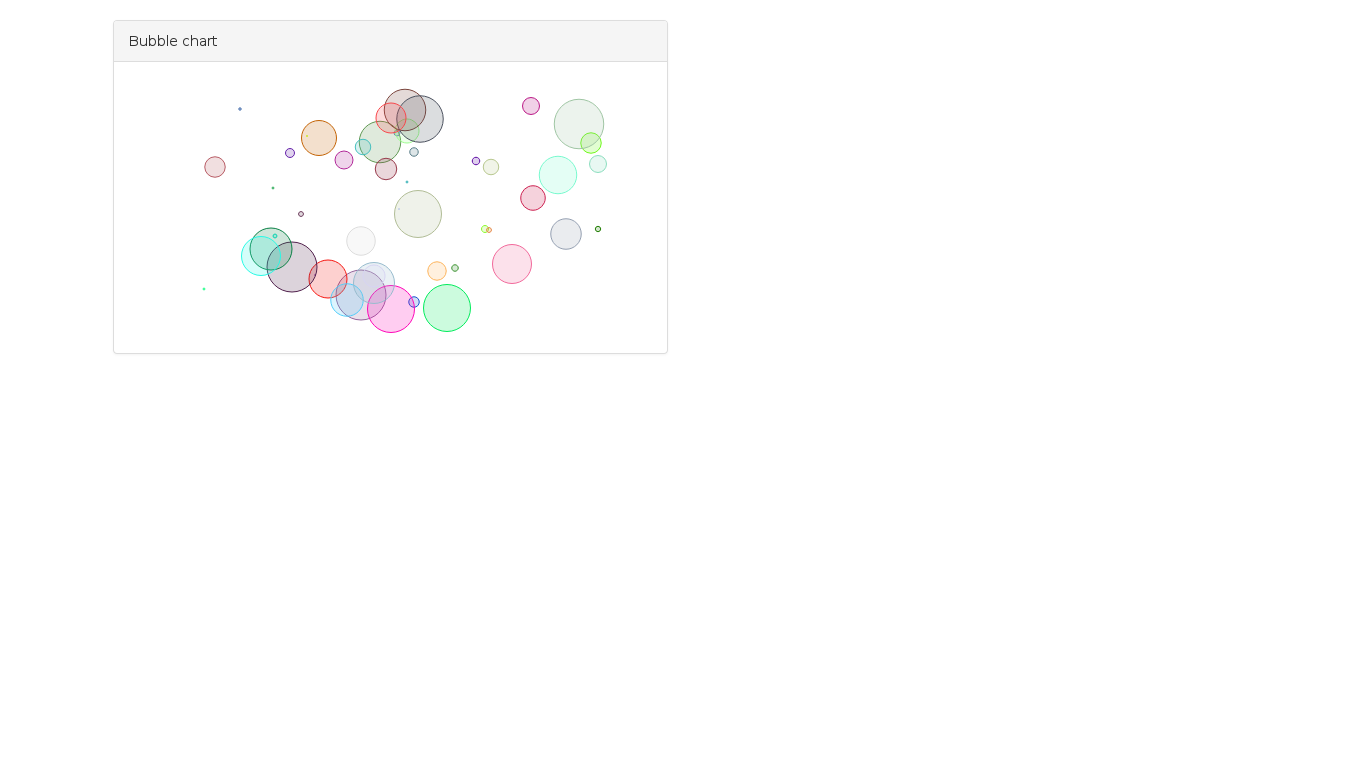

Supplement: Source Code [file NIHMS77548-supplement-Source_Code.zip › www/lib/angular-chart.js/test/fixtures/bubble.png]

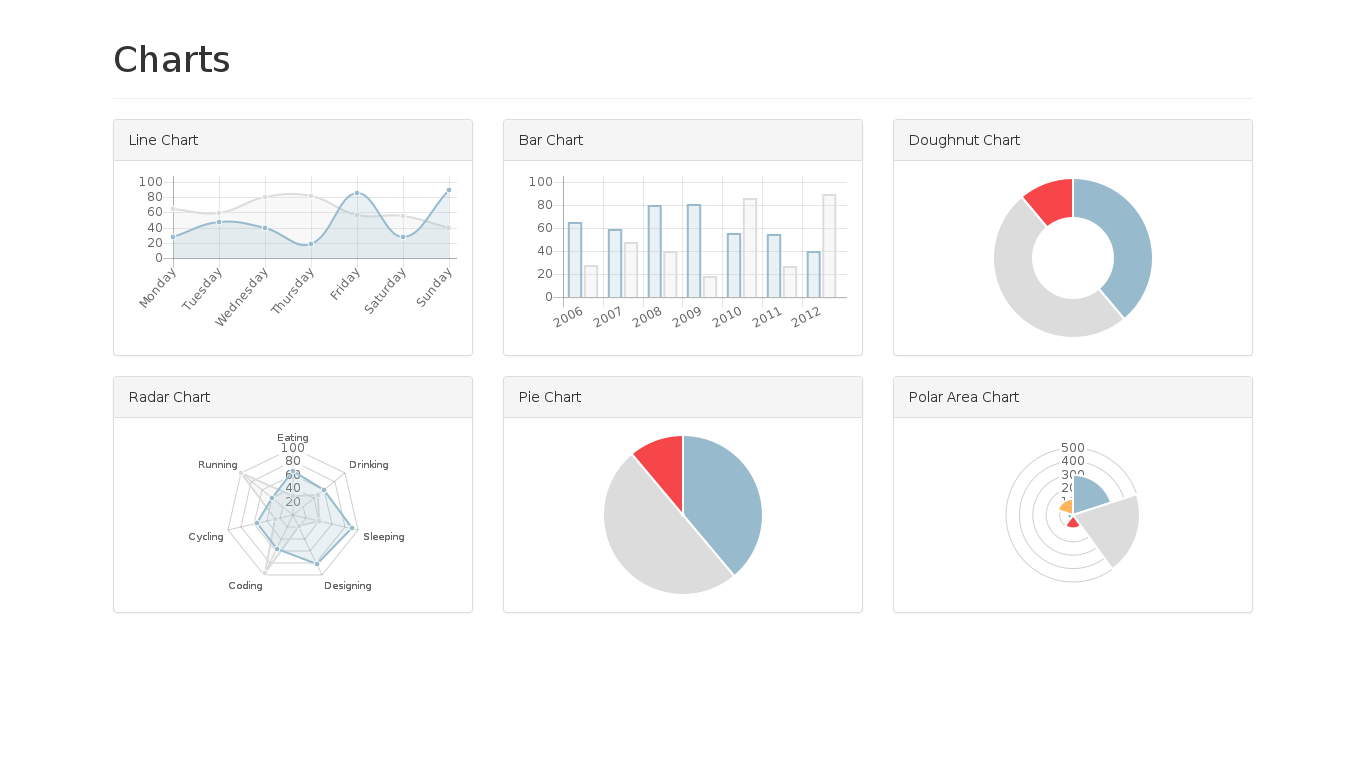

Supplement: Source Code [file NIHMS77548-supplement-Source_Code.zip › www/lib/angular-chart.js/test/fixtures/charts.png]

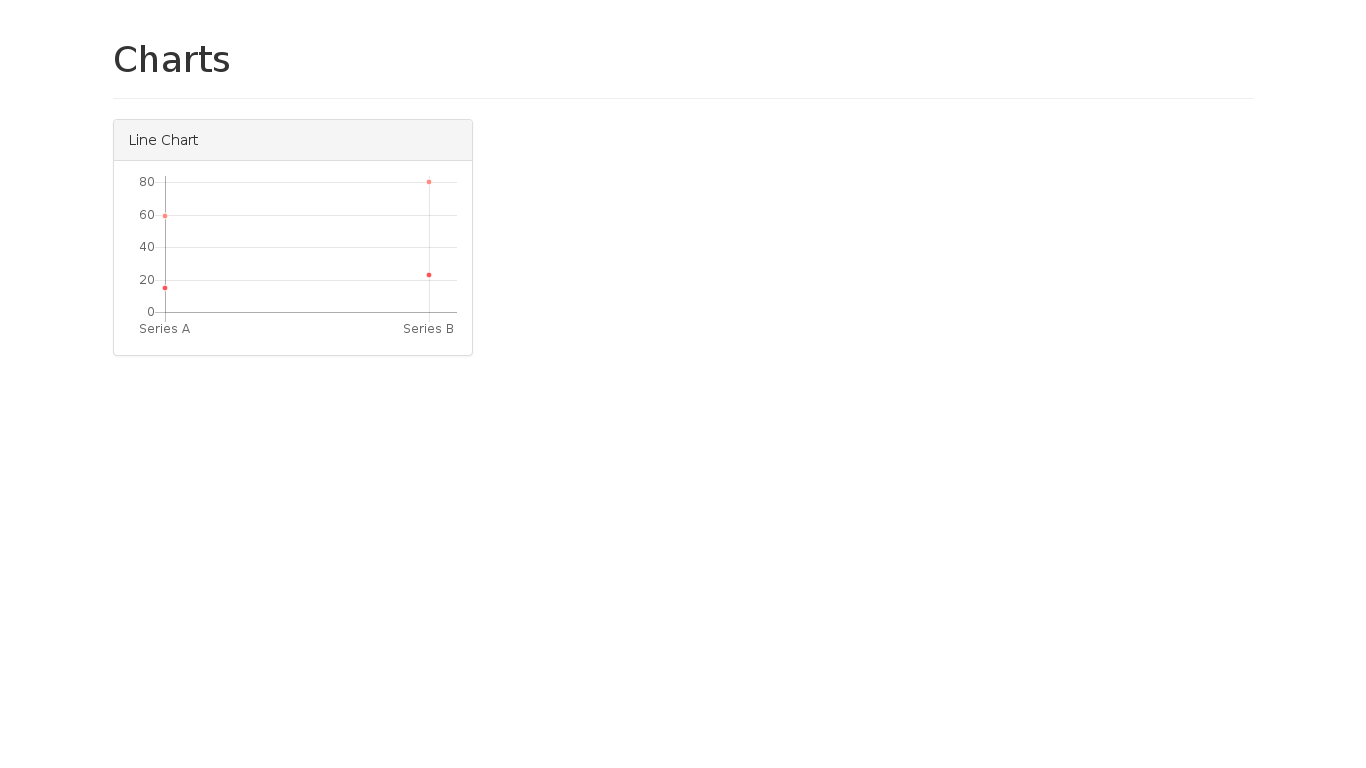

Supplement: Source Code [file NIHMS77548-supplement-Source_Code.zip › www/lib/angular-chart.js/test/fixtures/configure-line-chart.png]

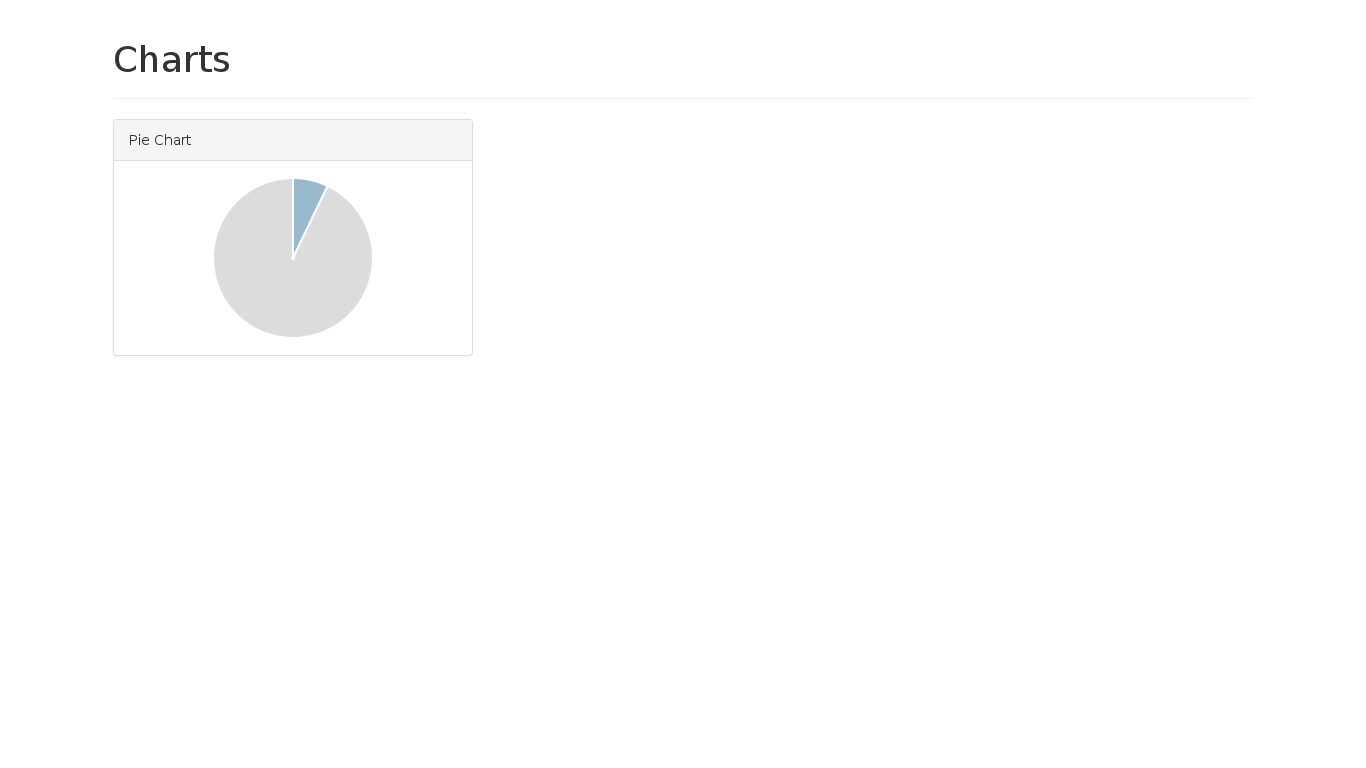

Supplement: Source Code [file NIHMS77548-supplement-Source_Code.zip › www/lib/angular-chart.js/test/fixtures/custom-directive.png]

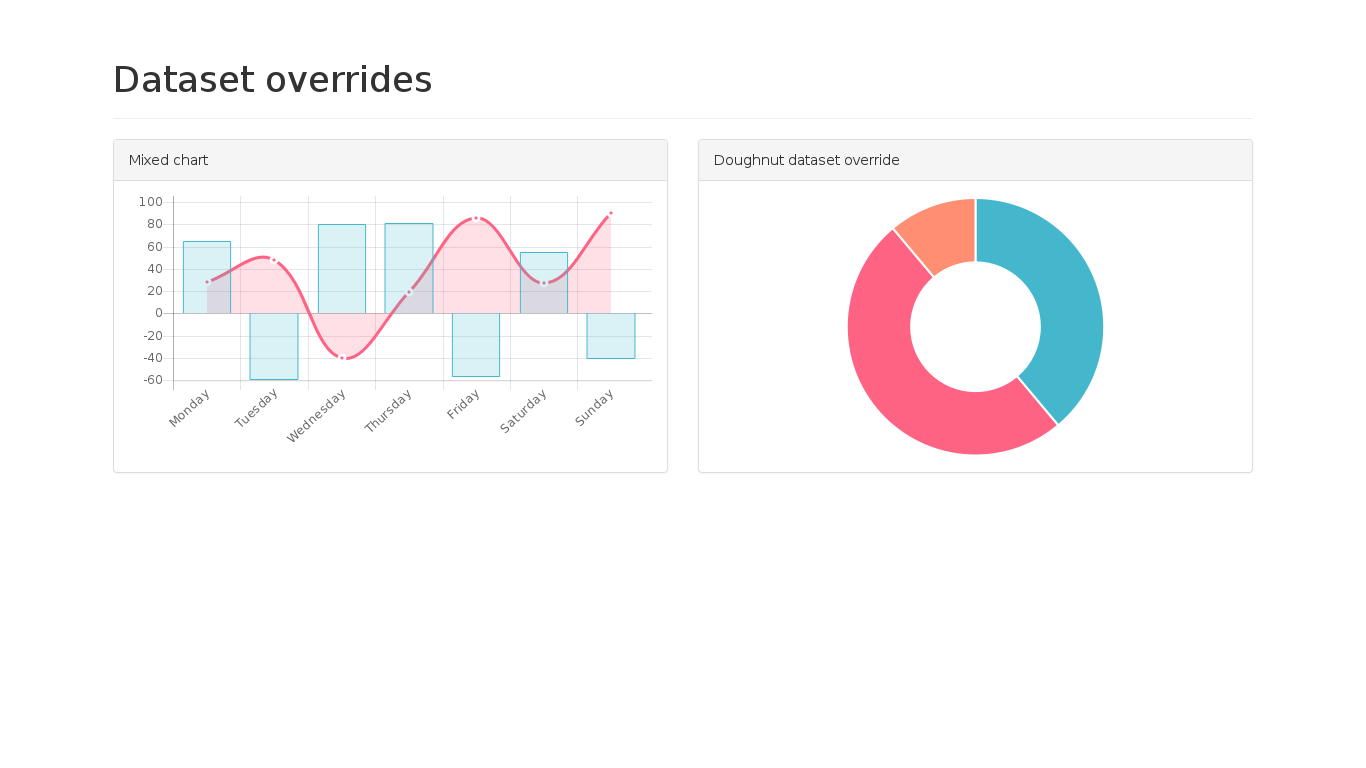

Supplement: Source Code [file NIHMS77548-supplement-Source_Code.zip › www/lib/angular-chart.js/test/fixtures/dataset-override.png]

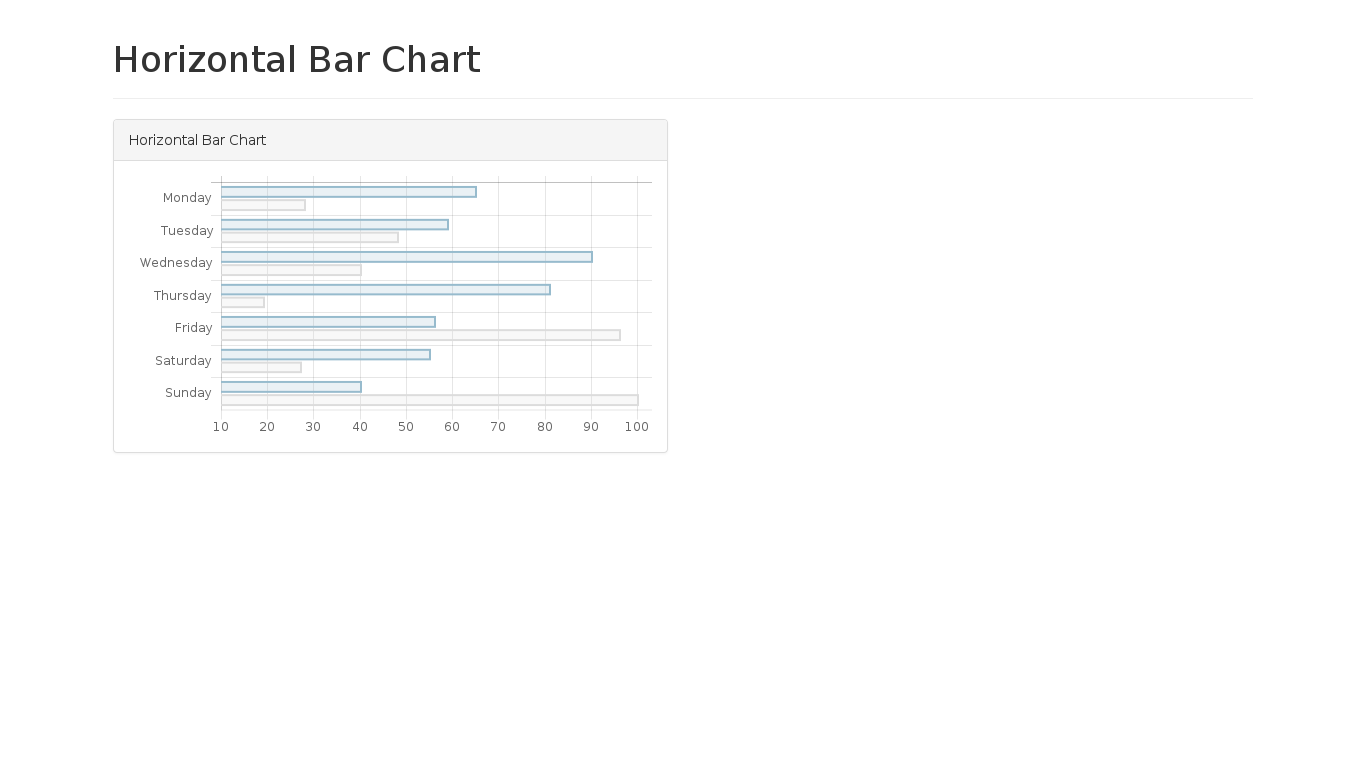

Supplement: Source Code [file NIHMS77548-supplement-Source_Code.zip › www/lib/angular-chart.js/test/fixtures/horizontal-bar-chart.png]

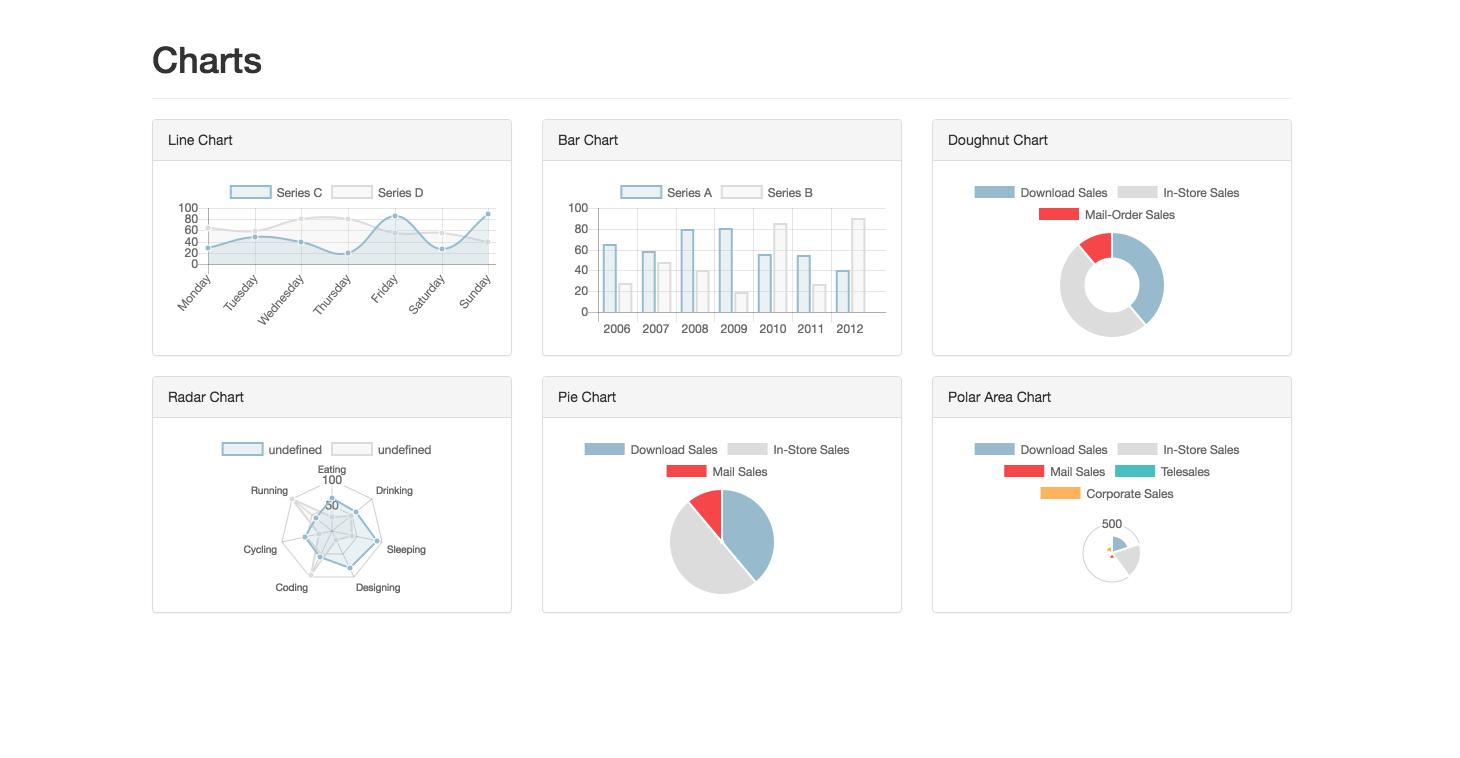

Supplement: Source Code [file NIHMS77548-supplement-Source_Code.zip › www/lib/angular-chart.js/test/fixtures/options-override.png]

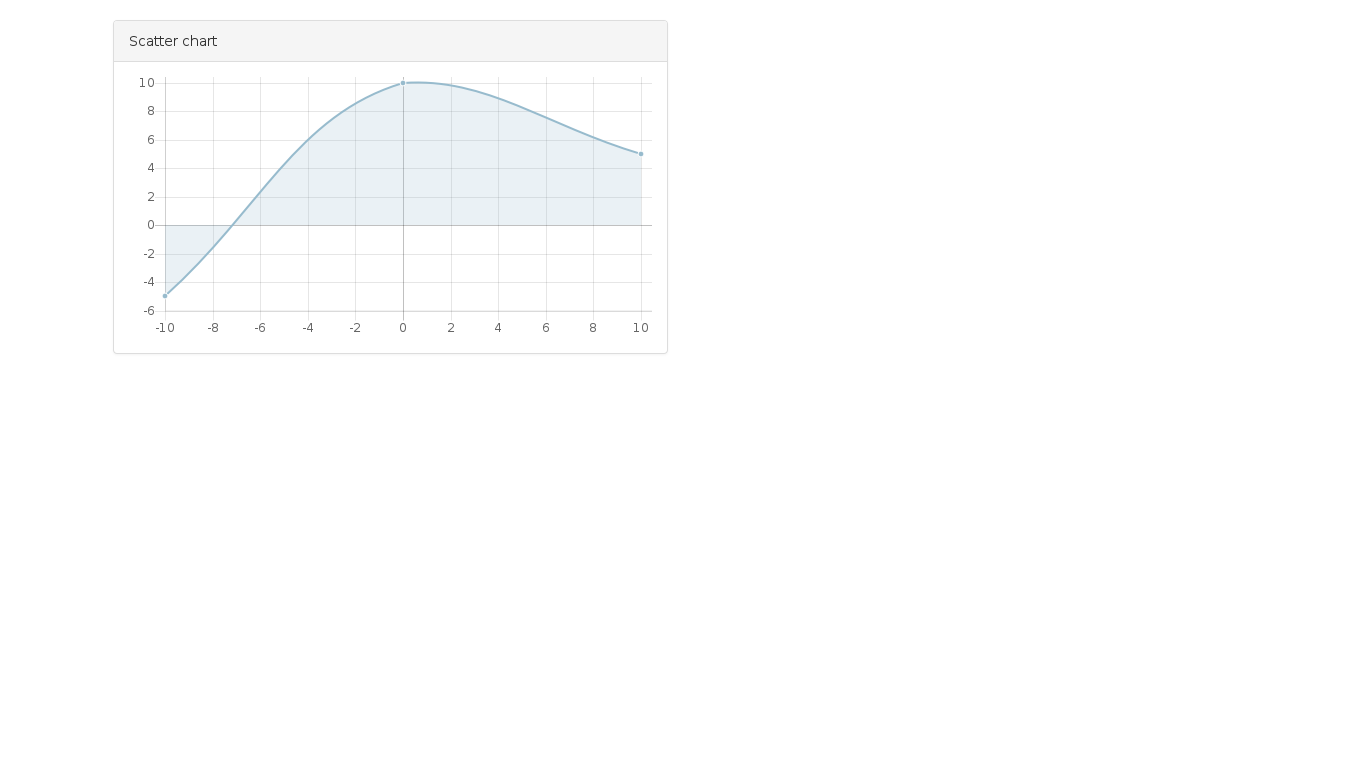

Supplement: Source Code [file NIHMS77548-supplement-Source_Code.zip › www/lib/angular-chart.js/test/fixtures/scatter.png]
