# Supplementary material for: InDEx: Open Source iOS and Android Software for Self-Reporting and Monitoring of Alcohol Consumption
Source: J Open Res Softw. Author manuscript; Available in PMC 2018 May 21. (PMC5961935; doi:10.5334/jors.207)
Supplement: Source Code [file NIHMS77548-supplement-Source_Code.zip › www/lib/angular-chart.js/tmp/bower_components/chart.js/samples/pie-customTooltips.html]

Pie Chart with Custom Tooltips
