# Supplementary material for: InDEx: Open Source iOS and Android Software for Self-Reporting and Monitoring of Alcohol Consumption
Source: J Open Res Softw. Author manuscript; Available in PMC 2018 May 21. (PMC5961935; doi:10.5334/jors.207)
Supplement: Source Code [file NIHMS77548-supplement-Source_Code.zip › www/templates/account/about/about.template.html]

{{ 'BACK\_ACCOUNT' | translate }}

InDEx is a project funded by the **Medical Research Council** to develop an alcohol intervention delivered via a web and mobile app with personalised text messaging. Our aim is to help people leaving the UK Armed Forces to reduce their drinking and to understand more about their patterns of drinking.

The InDEx app is being developed by **King's Centre for Military Health Research at King's College London** and **University of Liverpool**. The study is led by Dr Laura Goodwin (University of Liverpool) with Professor Nicola Fear managing contributions from the King's Centre for Military Health Research.

Dr Daniel Leightley (King's Centre for Military Health Research) leads the design, development and deployment of the InDEx architecture and ecosystem. Jo-Anne Puddephatt (University of Liverpool) is responsible for the analysis and data collection.

Other members of the team include Professor Matt Field (University of Liverpool), Professor Colin Drummond (King's College London, Department of Addictions), Professor Roberto Rona (King's Centre for Military Health Research), Dr Toktam Mahmoodi (King's College London, Department of Informatics) and Lt Col Norman Jones ARRC PhD (Academic Department of Military Mental Health).

Our project website is accessible via the following link: index-app.org.
