# Supplementary material for: InDEx: Open Source iOS and Android Software for Self-Reporting and Monitoring of Alcohol Consumption
Source: J Open Res Softw. Author manuscript; Available in PMC 2018 May 21. (PMC5961935; doi:10.5334/jors.207)
Supplement: Source Code [file NIHMS77548-supplement-Source_Code.zip › www/templates/account/account.template.html]

## {{vm.userData.firstname}} {{vm.userData.surname}}

Joined on {{vm.userData.createdAt | date:'dd MMMM yyyy'}}


{{'YOUR\_INFORMED\_CONSENT' | translate}}

{{'DRINKS\_FEEDBACK' | translate}}


## {{'DISCOVER' | translate}}


{{'HELP\_ARTICLES' | translate}}

{{'HELP\_TUTORIALS' | translate}}


{{'ABOUT\_INDEX' | translate}}


{{'LOGOUT' | translate}}
