# Supplementary material for: InDEx: Open Source iOS and Android Software for Self-Reporting and Monitoring of Alcohol Consumption
Source: J Open Res Softw. Author manuscript; Available in PMC 2018 May 21. (PMC5961935; doi:10.5334/jors.207)
Supplement: Source Code [file NIHMS77548-supplement-Source_Code.zip › www/templates/account/accountDisplay/accountDisplay.template.html]

{{ 'BACK\_ACCOUNT' | translate }}


## {{vm.userData.firstname}} {{vm.userData.surname}}

0{{vm.userData.smsmobile}}

{{vm.userData.email}}


{{ 'EDIT\_YOUR\_ACCOUNT' | translate }}

## {{ 'BADGES' | translate }}

## Drink Logger

text\_details


## Target setter

text\_details
