# Supplementary material for: InDEx: Open Source iOS and Android Software for Self-Reporting and Monitoring of Alcohol Consumption
Source: J Open Res Softw. Author manuscript; Available in PMC 2018 May 21. (PMC5961935; doi:10.5334/jors.207)
Supplement: Source Code [file NIHMS77548-supplement-Source_Code.zip › www/templates/account/consent/consent.template.html]

{{ 'BACK\_ACCOUNT' | translate }}

{{ 'ACCOUNT\_CONSENT\_INTRO1' | translate }} {{vm.userJoinDate | date:'dd MMMM yyyy'}}{{ 'ACCOUNT\_CONSENT\_INTRO2' | translate }}

{{ 'CONSENT\_1' | translate }}
{{ 'CONSENT\_2' | translate }}
{{ 'CONSENT\_3' | translate }}
{{ 'CONSENT\_4' | translate }}
{{ 'CONSENT\_5' | translate }}
{{ 'CONSENT\_6' | translate }}
{{ 'CONSENT\_7' | translate }}
{{ 'CONSENT\_8' | translate }}
{{ 'CONSENT\_9' | translate }}
{{ 'CONSENT\_10' | translate }}
{{ 'CONSENT\_11' | translate }}
{{ 'CONSENT\_12' | translate }}
