# Supplementary material for: InDEx: Open Source iOS and Android Software for Self-Reporting and Monitoring of Alcohol Consumption
Source: J Open Res Softw. Author manuscript; Available in PMC 2018 May 21. (PMC5961935; doi:10.5334/jors.207)
Supplement: Source Code [file NIHMS77548-supplement-Source_Code.zip › www/templates/account/walkthrough/walkthrough.template.html]

{{ 'BACK\_ACCOUNT' | translate }}


## Dashboard

Tutorial on how to use the dashboard


## Understanding your Goals

Tutorial on how to use the goals


## Setting a Goal

Tutorial on to set a goal


## Adding a Drink

Tutorial on how to add a drink


## Understanding your Diary

Tutorial on how to use the drinks diary
