# Supplementary material for: InDEx: Open Source iOS and Android Software for Self-Reporting and Monitoring of Alcohol Consumption
Source: J Open Res Softw. Author manuscript; Available in PMC 2018 May 21. (PMC5961935; doi:10.5334/jors.207)
Supplement: Source Code [file NIHMS77548-supplement-Source_Code.zip › www/templates/addDrinks/addDrinks.template.html]

{{vm.date | date : format : mediumDate}}


{{'ADD\_DRINKS\_ALCOHOL\_FREE' | translate}}

- {{vm.date | date : format : mediumDate}} {{ 'ADD\_DRINK\_TODAY\_FREE' | translate }}
- {{ 'ADD\_DRINK\_KEEP\_UP' | translate }}


{{ 'DRINK\_OPTIONS' | translate }}


{{ 'LOG' | translate }}
