# Supplementary material for: InDEx: Open Source iOS and Android Software for Self-Reporting and Monitoring of Alcohol Consumption
Source: J Open Res Softw. Author manuscript; Available in PMC 2018 May 21. (PMC5961935; doi:10.5334/jors.207)
Supplement: Source Code [file NIHMS77548-supplement-Source_Code.zip › www/templates/addDrinks/drinkInfo.template.html]

**{{vm.drink\_title}}**

{{ 'QUANTITY' | translate }}

**{{ 'ADD\_DRINK\_OPTIONAL' | translate }}**

Strength


{{ 'ADD\_DRINK\_COST' | translate }}


{{ 'CALORIES\_PER\_DRINK' | translate }}


{{ 'ADD\_DRINK\_WHO\_ARE\_YOU\_WITH' | translate }}

{{option}}


{{ 'ADD\_DRINK\_WHERE\_ARE\_YOU' | translate }}

{{option}}
