# Supplementary material for: InDEx: Open Source iOS and Android Software for Self-Reporting and Monitoring of Alcohol Consumption
Source: J Open Res Softw. Author manuscript; Available in PMC 2018 May 21. (PMC5961935; doi:10.5334/jors.207)
Supplement: Source Code [file NIHMS77548-supplement-Source_Code.zip › www/templates/addDrinks/drinkList.template.html]

- {{ 'BEER' | translate }}

- {{ 'WINE' | translate }}

- {{ 'CIDER' | translate }}

- {{ 'COCKTAIL' | translate }}

- {{ 'SPIRITS' | translate }}

- {{ 'ALCOPOPS' | translate }}
