# Supplementary material for: InDEx: Open Source iOS and Android Software for Self-Reporting and Monitoring of Alcohol Consumption
Source: J Open Res Softw. Author manuscript; Available in PMC 2018 May 21. (PMC5961935; doi:10.5334/jors.207)
Supplement: Source Code [file NIHMS77548-supplement-Source_Code.zip › www/templates/addDrinks/optionDrinks.template.html]

- {{data.drink\_name}} - {{data.drink\_measure}}
- {{ 'ADD\_DRINK\_ABV' | translate }} {{data.abv}}%
