# Supplementary material for: InDEx: Open Source iOS and Android Software for Self-Reporting and Monitoring of Alcohol Consumption
Source: J Open Res Softw. Author manuscript; Available in PMC 2018 May 21. (PMC5961935; doi:10.5334/jors.207)
Supplement: Source Code [file NIHMS77548-supplement-Source_Code.zip › www/templates/dashboard/dashboard.template.html]

# {{vm.daysUsing}}

{{ 'USING\_APP' | translate }}

## Welcome back {{vm.userData.preferred\_name}}!

## {{'YOUR\_ACTIVE\_GOALS' | translate}}

## {{goal.advice2}}{{goal.goal\_target}}{{goal.advice3}}

{{'YOUR\_ARE\_CURRENTLY' | translate}} {{goal.progress}} {{goal.option\_name\_lower}}

## {{'YOUR\_PROGRESS\_SO\_FAR' | translate}}

{{vm.total\_units}}

{{ 'UNITS\_CONSUMED' | translate }}  {{ 'THIS\_TIME\_LAST\_WEEK' | translate }}
{{ 'STARTED\_APP' | translate }}

{{vm.total\_drinks}}

{{ 'DRINKS\_CONSUMED' | translate }}
 {{ 'THIS\_TIME\_LAST\_WEEK' | translate }}
{{ 'STARTED\_APP' | translate }}

{{vm.total\_calories}}

{{ 'ALCOHOL\_CALORIES' | translate }}
 {{ 'THIS\_TIME\_LAST\_WEEK' | translate }}
{{ 'STARTED\_APP' | translate }}

{{vm.total\_exercise}}{{ 'MINUTES' | translate }}

{{ 'EQ\_RUNNING' | translate }}
 {{ 'THIS\_TIME\_LAST\_WEEK' | translate }}
{{ 'STARTED\_APP' | translate }}

{{vm.total\_burgers }}

{{ 'EQ\_BURGERS' | translate }}
 {{ 'THIS\_TIME\_LAST\_WEEK' | translate }}
{{ 'STARTED\_APP' | translate }}

£{{vm.total\_cost}}

{{ 'EST\_COSTS' | translate }}
 {{ 'THIS\_TIME\_LAST\_WEEK' | translate }}
{{ 'STARTED\_APP' | translate }}
