# Supplementary material for: InDEx: Open Source iOS and Android Software for Self-Reporting and Monitoring of Alcohol Consumption
Source: J Open Res Softw. Author manuscript; Available in PMC 2018 May 21. (PMC5961935; doi:10.5334/jors.207)
Supplement: Source Code [file NIHMS77548-supplement-Source_Code.zip › www/templates/drinks/drinks.template.html]

## {{ 'ADD\_DRINK\_HOW\_MANY' | translate }}


## {{ 'HOW\_MANY\_UNIT' | translate }}


## {{ 'MANY\_TOTAL\_CAL' | translate }}


## {{ 'HOW\_MUCH\_MONEY' | translate }}


{{ items.date | date:'fullDate'}}

## {{'ADD\_DRINKS\_ALCOHOL\_FREE' | translate}}

{{'CAL\_DRUNK' | translate}}: {{items.drink\_calories\_total}}

{{'LIKE\_COST' | translate}}: £{{items.drink\_price\_total}}

{{'TOTAL\_DRINKS' | translate}}: {{items.drink\_qty}}

{{'UNITS' | translate}}: {{items.drink\_units\_total}}

{{'EDIT' | translate}}
