# Supplementary material for: InDEx: Open Source iOS and Android Software for Self-Reporting and Monitoring of Alcohol Consumption
Source: J Open Res Softw. Author manuscript; Available in PMC 2018 May 21. (PMC5961935; doi:10.5334/jors.207)
Supplement: Source Code [file NIHMS77548-supplement-Source_Code.zip › www/templates/drinks/edit.template.html]

{{ 'BACK\_DIARY' | translate }}

## {{returnDrinkName(items.drink\_id)}}

{{ 'QUANTITY' | translate }}: {{items.drink\_qty}}

- Edit
- Delete
