# Supplementary material for: InDEx: Open Source iOS and Android Software for Self-Reporting and Monitoring of Alcohol Consumption
Source: J Open Res Softw. Author manuscript; Available in PMC 2018 May 21. (PMC5961935; doi:10.5334/jors.207)
Supplement: Source Code [file NIHMS77548-supplement-Source_Code.zip › www/templates/goals/goals.template.html]

### {{'REALISTIC\_GOAL' | translate }}

### {{ 'GOALS\_NOT\_YET' | translate }}

### {{ 'GOALS\_NOW' | translate }}

{{'SET\_GOAL' | translate}}

{{goal.progress}}

{{goal.option\_name\_lower}}

{{goal.desc}}. This goal lasts for {{goal.day\_length}} day(s)

Goal: {{goal.advice2}}{{goal.goal\_target}}{{goal.advice3}} Goal ends: {{goal.end\_date | date:'dd/MM/yy'}}

IF... {{goal.if}} THEN... {{goal.then}}...

### Previous Goals

{{goal.progress}}

{{goal.option\_name\_lower}}

Goal: {{goal.advice2}}{{goal.goal\_target}} {{goal.option\_name\_lower}}{{goal.advice3}}  You finished this goal on {{goal.end\_date | date:'dd/MM/yy'}}
