# Supplementary material for: InDEx: Open Source iOS and Android Software for Self-Reporting and Monitoring of Alcohol Consumption
Source: J Open Res Softw. Author manuscript; Available in PMC 2018 May 21. (PMC5961935; doi:10.5334/jors.207)
Supplement: Source Code [file NIHMS77548-supplement-Source_Code.zip › www/templates/goals/setGoals.template.html]

{{'GOAL\_ENDS' | translate}} {{vm.goalSet.end\_date | date:'dd/MM/yy'}}


# {{vm.goalData.desc}}: Step 1: {{vm.goalData.advice}}

{{$index+1}} {{vm.goalData.option\_name}}

## {{'GOALS\_TEXT\_BARRIER' | translate}}

## Step 2: IF...(the barrier)

{{i.question}}

## Step 3: THEN...(the solution)

{{i}}

## You have **{{vm.goalData.day\_length}}** day(s) to complete this goal


{{'SET\_GOAL' | translate }}
