# Supplementary material for: InDEx: Open Source iOS and Android Software for Self-Reporting and Monitoring of Alcohol Consumption
Source: J Open Res Softw. Author manuscript; Available in PMC 2018 May 21. (PMC5961935; doi:10.5334/jors.207)
Supplement: Source Code [file NIHMS77548-supplement-Source_Code.zip › www/templates/normative/normative.template.html]

{{ 'BACK\_ACCOUNT' | translate }}

### {{vm.user\_text\_status}}

### How is your drinking compared to those around you?

#### What does this mean?

#### The PURPLE line represents the general population and their drinking

#### The GREEN dashed line represents you and your drinking

### Here is how you compare based on gender...


{{vm.people\_count\_text}}

### This is how many calories you drink each week Click to find the alcohol slice...

Here are your calories each week...

{{vm.calSumm}}. That is a total of {{vm.burger\_num}} McDonald's Big Mac Burgers!


### Here is how much you are spending on alcohol...

We estimate that you spend on average £{{vm.weeklyCost}} a week on alcohol

### What next?

- You can use this information to help you monitor your drinking. You will have good days, and bad. But try to remember the following helpful advice:
- You can still enjoy a drink. But try to remember the following advice:
- **Make a plan.**
- **Set a budget.**
- **Let family/friends know.**
- **Take it a day at a time.**
- **Make it a smaller one.**
- **Have a lower-strength drink.**
- **Stay hydrated.**
- Remember **Take a break.**


{{vm.source\_summary}}
