# Supplementary material for: InDEx: Open Source iOS and Android Software for Self-Reporting and Monitoring of Alcohol Consumption
Source: J Open Res Softw. Author manuscript; Available in PMC 2018 May 21. (PMC5961935; doi:10.5334/jors.207)
Supplement: Source Code [file NIHMS77548-supplement-Source_Code.zip › www/templates/outside/register/register.template.html]

Back


1 {{ 'CONSENT' | translate }}
2 {{ 'PROFILE' | translate }}
3{{ 'MOBILE\_VER\_VALIDATE' | translate }}

## Welcome

**InDEx** is a project funded by the **Medical Research Council** to develop an alcohol intervention delivered via a hybrid web and mobile app. Our aim is to help people leaving the UK Armed Forces to reduce their drinking and to understand more about their patterns of drinking.

To enable us to conduct our research we will need you to complete a secure online registration process. This will generate a account that is unique to you.

{{ 'START\_REGISTER' | translate }}
