# Supplementary material for: InDEx: Open Source iOS and Android Software for Self-Reporting and Monitoring of Alcohol Consumption
Source: J Open Res Softw. Author manuscript; Available in PMC 2018 May 21. (PMC5961935; doi:10.5334/jors.207)
Supplement: Source Code [file NIHMS77548-supplement-Source_Code.zip › www/templates/outside/register/step2.template.html]

{{ 'REGISTER\_FIRST\_NAME' | translate }}


{{ 'REGISTER\_SURNAME' | translate }}


{{ 'REGISTER\_EMAIL' | translate }}


{{ 'REGISTER\_PREFFERED\_NAME' | translate }}


{{ 'REGISTER\_GENDER' | translate }}

{{ 'REGISTER\_GENDER\_MALE' | translate }}
{{ 'REGISTER\_GENDER\_FEMALE' | translate }}

{{ 'REGISTER\_MOBILE' | translate }}
+44

{{ 'REGISTER\_FIRST\_NAME\_ERROR' | translate }}

\* {{ 'REGISTER\_FIRST\_NAME\_ERROR\_REQUIRED' | translate }}

{{ 'REGISTER\_SURNAME\_ERROR' | translate }}

\* {{ 'REGISTER\_SURNAME\_ERROR\_REQUIRED' | translate }}

\* {{ 'REGISTER\_EMAIL\_ERROR\_REQUIRED' | translate }}

\* {{ 'REGISTER\_EMAIL\_ERROR\_TYPE' | translate }}

\* {{ 'REGISTER\_PREFFERED\_NAME\_ERROR\_REQUIRED' | translate }}

{{ 'REGISTER\_PREFFERED\_NAME\_ERROR\_MIN' | translate }}

{{ 'REGISTER\_PREFFERED\_NAME\_ERROR\_MAX' | translate }}

\* {{ 'REGISTER\_GENDER\_ERROR\_REQUIRED' | translate }}

{{ 'REGISTER\_MOBILE\_MIN' | translate }}

{{ 'REGISTER\_MOBILE\_MAX' | translate }}

\* {{ 'REGISTER\_MOBILE\_REQUIRED' | translate }}

\* {{ 'REGISTER\_MOBILE\_NUMBER' | translate }}

{{ 'REGISTER' | translate }}
