# Supplementary material for: InDEx: Open Source iOS and Android Software for Self-Reporting and Monitoring of Alcohol Consumption
Source: J Open Res Softw. Author manuscript; Available in PMC 2018 May 21. (PMC5961935; doi:10.5334/jors.207)
Supplement: Source Code [file NIHMS77548-supplement-Source_Code.zip › www/templates/outside/register/step3.template.html]

## {{ 'MOBILE\_VER\_SMS' | translate }}

### {{ 'MOBILE\_VER\_ENTER\_BELOW' | translate }} +44 {{registerDataForm.smsmobile}}


Code not the required length

{{ 'MOBILE\_VER\_VALIDATE' | translate }}
