# Supplementary material for: InDEx: Open Source iOS and Android Software for Self-Reporting and Monitoring of Alcohol Consumption
Source: J Open Res Softw. Author manuscript; Available in PMC 2018 May 21. (PMC5961935; doi:10.5334/jors.207)
Supplement: Source Code [file NIHMS77548-supplement-Source_Code.zip › www/templates/screening/registration/registration.template.html]

# {{vm.screeningTitle}}


{{'SCREENING\_INFO' | translate}}

Your progress...

 

{{ vm.question }}

You can select up to **{{ vm.num\_response }}** responses.

- {{option}}

{{option}}


Your Score: {{dataObject[vm.type\_id[0]]}}

*0*

*10*


{{'NEXT\_TEXT' | translate}}


{{'LOG' | translate}}
