# Supplementary material for: InDEx: Open Source iOS and Android Software for Self-Reporting and Monitoring of Alcohol Consumption
Source: J Open Res Softw. Author manuscript; Available in PMC 2018 May 21. (PMC5961935; doi:10.5334/jors.207)
Supplement: Source Code [file NIHMS77548-supplement-Source_Code.zip › www/templates/support/support.template.html]

### Organisations to contact if you are worried about your drinking:

**Drinkline**

Drinkline runs a free, confidential helpline for people who are concerned about their drinking, or someone else's. The purpose of the Drinkline service is to offer free, confidential, accurate and consistent information and advice to callers who are concerned
about their own or someone else`s drinking regardless of the caller`s age, gender, sexuality, ethnicity or spirituality.

**Helpline:** 0300 123 1110

**Addaction**

A UK wide treatment agency, helping individuals, families and communities to manage the effects of drug and alcohol misuse.

**Website:**  http://www.addaction.org.uk/

**Al-Anon**

Al-Anon is worldwide and offers support and understanding to the families and friends of problem drinkers.

**Helpline:** 0207 4030 888

**Samaritans**

Provides confidential non-judgmental emotional support, 24 hours a day for people who are experiencing feelings of distress, despair or suicide.

**Helpline:** 08457 909 090

### Organisations which offer support specifically for veterans:

**Armed Forces Community Directory**

Information on a range of available health, education, employment, housing, legal and welfare services for members of the Armed Forces community, including veterans by geographical area.

**Website:**  https://www.afcom.com

**Civvy Street**

Free online service supported by The Royal British Legion to help Service personnel transition into civilian life. Provides information, interactive services, resources and links to employers and service organisations.

**Website:**  https://www.civvystreet.org

**Royal British Legion**

The Royal British Legion helps serving members of the Armed Forces, ex-Service men and women (veterans), their families and dependants all year-round.

**Helpline:** 08457 725 725

**Veteran’s Association UK**

The Veteran's Association UK's provides to veterans, serving personnel and their families in accessing pensions, benefits, housing, treatment for mental or other health issues.

**Helpline:** 01282 219 391
